# N6-methyladenosine RNA modification promotes viral genomic RNA stability and infection

**DOI:** 10.1038/s41467-022-34362-x

**Published:** 2022-11-02

**Authors:** Tianye Zhang, Chaonan Shi, Haichao Hu, Zhuo Zhang, Ziqiong Wang, Zhiqing Chen, Huimin Feng, Peng Liu, Jun Guo, Qisen Lu, Kaili Zhong, ZhiHui Chen, Jiaqian Liu, Jiancheng Yu, Jianping Chen, Feng Chen, Jian Yang

**Affiliations:** 1 State Key Laboratory for Quality and Safety of Agro-products, Key Laboratory of Biotechnology in Plant Protection of Ministry of Agriculture and Rural Affairs and Zhejiang Province, Institute of Plant Virology, Ningbo University, Ningbo, 315211 China; 2 National Key Laboratory of Wheat and Maize Crop Science/Collaborative Innovation Center of Henan Grain Crops/Agronomy College, Henan Agricultural University, Zhengzhou, 450000 China; 3 Hunan Plant Protection Institute, Hunan Academy of Agricultural Sciences, Changsha, 410000 China; 4 University of Dundee, School of Life Sciences, Dow Street, Dundee, DD1 5EH UK; 5 Zhejiang Engineering Research Center of Advanced Mass spectrometry and Clinical Application, Ningbo University, Ningbo, 315211 China

**Keywords:** Plant genetics, Agricultural genetics, Biotic, Agricultural genetics

## Abstract

Molecular manipulation of susceptibility (*S*) genes that are antipodes to resistance (*R*) genes has been adopted as an alternative strategy for controlling crop diseases. Here, we show the *S* gene encoding *Triticum aestivum* m^6^A methyltransferase B (TaMTB) is identified by a genome-wide association study and subsequently shown to be a positive regulator for wheat yellow mosaic virus (WYMV) infection. TaMTB is localized in the nucleus, is translocated into the cytoplasmic aggregates by binding to WYMV NIb to upregulate the m^6^A level of WYMV RNA1 and stabilize the viral RNA, thus promoting viral infection. A natural mutant allele TaMTB-SNP176C is found to confer an enhanced susceptibility to WYMV infection through genetic variation analysis on 243 wheat varieties. Our discovery highlights this allele can be a useful target for the molecular wheat breeding in the future.

## Abstract (web summary)

Wheat production is threatened by wheat yellow mosaic virus (WYMV). Here, via genome-wide association study, the authors report that a putative methyltransferase B positively regulates WYMV infection through enhancing viral genomic RNA stability by N6-methyladenosine RNA modification.

## Introduction

Wheat (*Triticum aestivum*) is one of the most important staple cereal crops in the world. Common wheat is allohexaploid with three sub-genomes (AABBDD), its large size of genome (~ 16 Gb) and high degree of duplication poses a great challenge for gene cloning. However, the release of some wheat reference genomes (http://www.wheatgenome.org/) has now provided an important platform for gene identification. Wheat production in east Asia has long been threatened by wheat yellow mosaic virus (WYMV) disease. Infected plants have retarded growth with mosaic or yellow-striped leaves and consequently a reduced yield^1^. Up to 20–70% yield losses were reported in some wheat growing regions in China^2,3^. Molecular breeding of resistant cultivars is the most effective and environment friendly way to control the disease^4^. Dominant resistance (*R*) genes are often the preferred breeding targets for molecular manipulation but such resistance is often race-specific and easily overcome by spontaneous mutation of the pathogen^5^. With the development of genome editing technologies, the susceptibility (*S*) genes required for pathogen infection have become increasingly attractive targets for editing to generate new resistant genotypes^4^. Function-losing mutations in *S* genes substantially reduce the compatibility between hosts and pathogens, thus provide a durable and broad-spectrum resistance to many pathogen isolates. For examples, a programmed genome editing of a *S* gene, Mildew *resistance locus O* (*MLO*), in wheat conferred a robust broad-spectrum powdery mildew resistance without adverse effects on crop growth and yields^6^. Naturally-occurring variation in the recessive *ROD1* alleles contributes to subspecies-specific disease resistance in rice without yield penalty^7^. It has therefore become highly desirable to identify and select susceptibility genes for molecular breeding of resistant wheat cultivars.

WYMV has a bipartite positive-sense RNA genome and is classified in genus *Bymovirus*, family *Potyviridae*. RNA1 encodes a polyprotein which is processed into eight different proteins, including the nuclear inclusion “b” protein (NIb) which function as an RNA-dependent RNA polymerase and is important for virus replication^8^. RNA2 also encodes a polyprotein which produces two mature proteins, P1 and P2^9^. To date, several genes or quantitative trait locus (QTL) loci have been shown to confer resistance against WYMV in wheat, including *YmYF*, *YmNM*, *QYm.njau-5A.1*, *QYm.njau-3B.1* and *QYm.njau-7B.1*^2,10,11^, but all of these are dominant.

The m^6^A methylation is one of the most prevalent internal post-transcriptional modification of RNA in eukaryotes^12,13^. The addition of m^6^A onto mRNA, which occurs at the consensus motif DRAmCH (where D = G/A/U, R = G > A, and H = U/C/A), has been reported to regulate several processes of mRNA metabolism including mRNA stability^14^, translation^15^, nuclear export^16^ and exon splicing^17^. The reversible m^6^A modification is controlled by three main components of the m^6^A methylation machinery called writers, erasers and readers. In plants, a number of proteins belonging to these three groups have been reported. Demethylases as erasers mainly belong to the AlkB family of Fe (II)/α-ketoglutarate dependent dioxygenases like ALKBH9B and ALKBH10B^18,19^. The m^6^A readers contain a YTH domain and bind to m^6^A-modified mRNAs to implement the biological function. CPSF30 and ECT2 have been characterized as m^6^A readers^20,21^. One of the earliest discovered writers in Arabidopsis was MTA (METTL3 human homolog protein)^22^ and subsequently, MTB (METTL14), FIP37 (WTAP), VIRILIZER, HAKAI and FIONA1 have also been shown to be m^6^A writers in plants^23–25^. In Arabidopsis, heterodimerization of MTA and MTB has been reported^23^. It has recently been shown that strawberry MTA interacts with MTB and RNA interference of these down-regulates the m^6^A level of series transcripts^26^. Apple MTA also interacts with MTB but methyltransferase activity depends on the presence of both MTA and MTB^27^. Although MTA has been studied in detail, its protein partner, MTB has been characterized less intensively and its function in plants remains largely unknown^13,28^. As an orthologue of METTL14, MTB has been described as a probable non-catalytic subunit of the N^6^-methyltransferase complex while one study suggested that both METTL3 and METTL14 have methyltransferase activity^29^. Although a later study suggested that METTL14 does not catalyze methyl-group transfer but provides an RNA-binding scaffold, the authors could not exclude the possibility that METTL14 possesses MTase activity under certain conditions^30^. Therefore, it is important to study the function of MTB in plants.

RNA viruses have a limited capacity to encode proteins and no viral-encoded m^6^A methyltransferase has been reported. However, increasing evidence indicates that m^6^A is present in viral RNA, which must be added by the host m^6^A methyltransferases. The m^6^A are involved in viral life cycle, regulating viral infection and also participating in host antiviral innate immunity^31,32^. the m^6^A sites are found in the HA coding region, enhancing HA mRNA expression and promoting Influenza A Virus infection^33^. Enterovirus 71 (EV71) genomic RNA is modified by m^6^A which promotes EV71 replication^34^. Hepatitis B virus X protein recruits METTL3/14 onto both the HBV minichromosome and the host PTEN chromosomal locus to add m^6^A modification, and then the added m^6^A in the 5’ or 3’ epsilon stem loop regulates both the stability of viral transcripts and the reverse transcription of pre-genomic RNA, while the added m^6^A destabilizes PTEN mRNA leading to immune evasion^35,36^. In addition, two studies both indicated that m^6^A modification in viral RNA enables viral RNA to escape recognition by host antiviral immunity^37,38^. Reduction of m^6^A in SARS-CoV-2 RNA increases RIG-I binding and enhances the downstream innate immune signaling pathway and inflammatory gene expression^39^. On the other hand, the m^6^A machinery negatively regulates HCV and ZIKV infection^40,41^. Among plant viruses, however, there have been very few studies on m^6^A. *Arabidopsis* ALKBH9B can bind alfalfa mosaic virus (AMV) RNA to regulate infection negatively by its demethylase activity^18,42^. Recently, m^6^A modification has been reported in rice infected by rice stripe virus (RSV) and rice black-streaked dwarf virus (RBSDV) and this influenced the transcription level of genes involved in antiviral pathways^43^. Host m^6^A modification level is also significantly altered after infection by tobacco mosaic virus (TMV) or WYMV^44,45^. However, the association between m^6^A and plant RNA viruses and the mechanism by which m^6^A regulates viral infection are still largely unknown.

In this work, a WYMV resistance locus is identified on chromosome 4B through GWAS and QTL mapping. Subsequently, a transcriptomic analysis of genes in this locus is undertaken to identify a candidate gene encoding methyltransferase B (TaMTB) in wheat. Through overexpressing or gene silencing experiments, we show that TaMTB positively regulates WYMV infection. TaMTB may be a m^6^A methyltransferase that is translocated to cytoplasmic punctate structures by interacting with WYMV NIb protein. We then identify a m^6^A site (A^6800^) in WYMV RNA1, which is upregulated by TaMTB. Mutational inactivation of this site decreases its stability and inhibited viral infection. We, therefore, propose that the allele TaMTB (176 ^A^) could be used for molecular breeding of wheat resistant to WYMV.

## Results

### TaMTB identified by GWAS and QTL is a positive regulator of WYMV infection in wheat

**Fig. 1:**
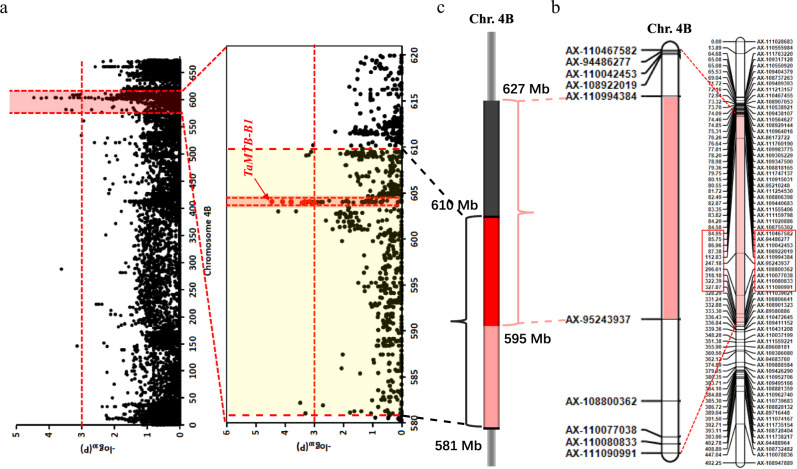
The *qWYM.hau-4B* was co-located by GWAS and QTL. **a** A WYMV candidate interval of 581.0-610.2 Mb on 4B was identified by genome-wide association study (GWAS) in 243 Chinese wheat accessions. The red horizontal line indicates the genome-wide significance threshold. Red background area indicates the candidate region (581.0-610.2 Mb) on 4B. The red dots above the threshold line represent significant SNPS located in the region of *TaMTB*. **b** LOD contours for quantitative trait loci (QTL) *qWYM.hau-4B* for WYMV resistance on 4B identified by inclusive composite interval mapping (ICIM) in the Xianyangdasui/Xinmai208 F_8_ RIL population. The red rectangle corresponds to the pink interval and represents the enlargement of that interval. **c** Co-location analysis of GWAS and QTL showing that *qWYM.hau-4B* was narrowed into an interval of 595-610 Mb. Red interval (595-610 Mb) represent the GWAS and QTL co-localization interval.

A total of 243 Chinese wheat accessions were used for genome-wide association study (GWAS) to identify WYMV resistance loci using the Wheat 660 K SNP array. Results of phenotypic investigation for three consecutive years showed that 54.7–81.5% of the test wheat accessions showed the infection type (IT) ≥ 3 (Supplementary Fig. 1), and correlation analyses showed that coefficient of correlation among three years ranged from 0.84 to 0.94. GWAS results revealed that significant SNPs were distributed on all chromosomes (Supplementary Fig. 2), and that 14 of them are located on 4B, mainly concentrated in the interval of 581.0–610.2 Mb (Fig. 1a, Supplementary Table 1). Furthermore, linkage mapping using the F_8_ RIL (recombinant inbred lines) population Xianyangdasui/Xinmai208 detected a total of 10 QTLs for WYMV distributed on 1 A, 1B, 2 A, 2D, 3B, 3D, 4 A, 4B, 4D and 5D. Of the 10 QTLs, *qWYM.hau-4B* flanked by markers AX-110994384 (627 Mb) and AX-95243937 (595 Mb) on 4B were also identified by GWAS (Fig. 1b). Combination analysis of GWAS and QTL showed that the *qWYMV.hau-4B* were narrowed into an interval of 595–610 Mb covering 129 annotated genes (Fig. 1c) in the genome database of Chinese Spring. Bulked segregant RNA-Seq (BSR) indicated that 31 of the 129 genes were differentially expressed in WYMV-resistant and WYMV-susceptible pools (Supplementary Data 1), and 20 of them were relatively high expression in wheat roots and stem [http://202.194.139.32/] (Supplementary Data 1). Sequencing results showed that 9 of the 20 genes had non-synonymous or deteriorated variation in the association panel (Supplementary Data 1, 2). Then, re-running GWAS after adding polymorphic sites of the 9 genes revealed that only *TaMTB-B1* was significantly associated with WYMV resistance (Fig. 1a, Supplementary Table 1). These results suggested that *TaMTB-4B* might played a key role in regulating WYMV resistance.

**Fig. 2:**
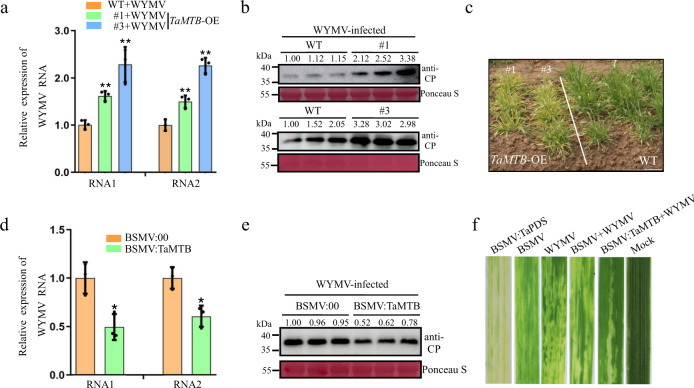
*TaMTB* positively regulates WYMV infection. **a** The accumulation of WYMV RNA1 and RNA2 in the WYMV-infected *TaMTB* transgenic (*TaMTB*-OE) or wild type (WT) plants as determined by qPCR using *CP* and *P2* gene-specific primers. #1, 3 are two independent transgenic lines of *TaMTB*-OE. Values are means ± SD (two-sided *t* test, *n*  =  3, *P*  =  0.0011, 0.0045, 0.0085, 0.0025, respectively). **P  <  0.01. **b** Detection of WYMV CP accumulation in WT or two *TaMTB*-OE lines plants by western blot using a CP-specific antibody. Three times each experiment was repeated independently with similar results. **c** Assessment of *TaMTB*-OE plants for disease resistance in a virus-contaminated nursery at Yangzhou, Jiangsu Province in 2022. WT represents *Fielder* plants. **d** WYMV RNAs accumulation in wheat plants co-infected with WYMV and BSMV:TaMTB or WYMV and BSMV:00. Total RNA from BSMV:00 and WYMV co-infected plants (BSMV:00) were used as negative controls. Values are means ± SD (two-sided *t* test, *n*  =  3, *P*  =  0.0128, 0.0189, respectively). *P  <  0.05. **e** Detection of *WYMV CP* accumulation in WYMV-infected BSMV:00 and BSMV: TaMTB plants by western blot using a CP-specific antibody. Three times each experiment was repeated independently with similar results. **f** Phenotypes in the fourth leaves of the plants inoculated with phosphate buffered saline (Mock), BSMV, WYMV, BSMV:PDS, BSMV + WYMV and BSMV:TaMTB+WYMV, respectively. Ponceau staining (Ponceau S) shows equal protein loadings in each lane. *TaCDC* was used as the internal control gene. Source data are provided as a Source Data file.

To investigate the function of *TaMTB* modulating WYMV resistance, we cloned it from wheat genome and overexpressed it with Flag tag in the ‘Fielder’ wheat. *TaMTB*-OE (*TaMTB*-overexpressed) lines were obtained and confirmed by a Western blot in T_0_ lines (Supplementary Fig. 3a) and tested again in T_2_ lines (Supplementary Fig. 3b). Subsequently, T_2_ transgenic plants *TaMTB*-OE were inoculated with RNA transcripts of WYMV and tested for WYMV RNA accumulation at 7 dpi by qRT-PCR. WYMV RNA accumulation was significantly higher in *TaMTB*-OE lines than in wild-type (Fielder) plants (Fig. 2a), and correspondingly greater amounts of viral coat protein were confirmed by the Western blot (Fig. 2b). We also observed that the mosaic symptoms in WYMV-inoculated *TaMTB*-OE plants were more severe than in WYMV-infected WT plants (Fig. 2c), suggesting that TaMTB acts as a host factor to promote WYMV infection.

To further investigate the effects of TaMTB on WYMV infection, we tried to generate a *TaMTB* knockout mutant through CRISPR-Cas9 but failed as *TaMTB* knockout plants turned out to be embryonic lethal. We, therefore, used barley stripe mosaic virus (BSMV) vectors to knock-down *TaMTB*. Two recombinant plasmids, BSMV:TaMTB and BSMV:TaPDS (phytoene desaturase gene, acted as a positive control), were constructed. Wheat seedlings were then inoculated with WYMV, BSMV, BSMV:TaPDS, BSMV:TaMTB, BSMV + WYMV or BSMV:TaMTB+WYMV. After 14 dpi, the infection of WYMV and BSMV were respectively confirmed through RT-PCR (Supplementary Fig. 4a) and the silencing of *TaMTB* in wheat seedlings inoculated with BSMV:TaMTB+WYMV was also confirmed (Supplementary Fig. 4b). Furthermore, WYMV RNAs accumulation in seedlings inoculated with BSMV:TaMTB+WYMV was significantly lower than in the control BSMV:00+WYMV (Fig. 2d). The accumulation of WYMV CP protein was also decreased in TaMTB silenced wheat than in BSMV:00 plants (Fig. 2e). In addition, at 14 dpi the systemically infected leaves of wheat seedlings inoculated with BSMV:TaPDS exhibited a typical photobleaching phenotype, whereas the leaves of plants inoculated with BSMV:TaMTB+WYMV displayed milder mosaic symptoms than the control plants inoculated with BSMV or BSMV + WYMV (Fig. 2f). These results indicate that TaMTB plays a positive regulatory role in WYMV infection in wheat.

### *TaMTB* is recruited into the cytoplasmic aggregates by interacting with NIb

**Fig. 3:**
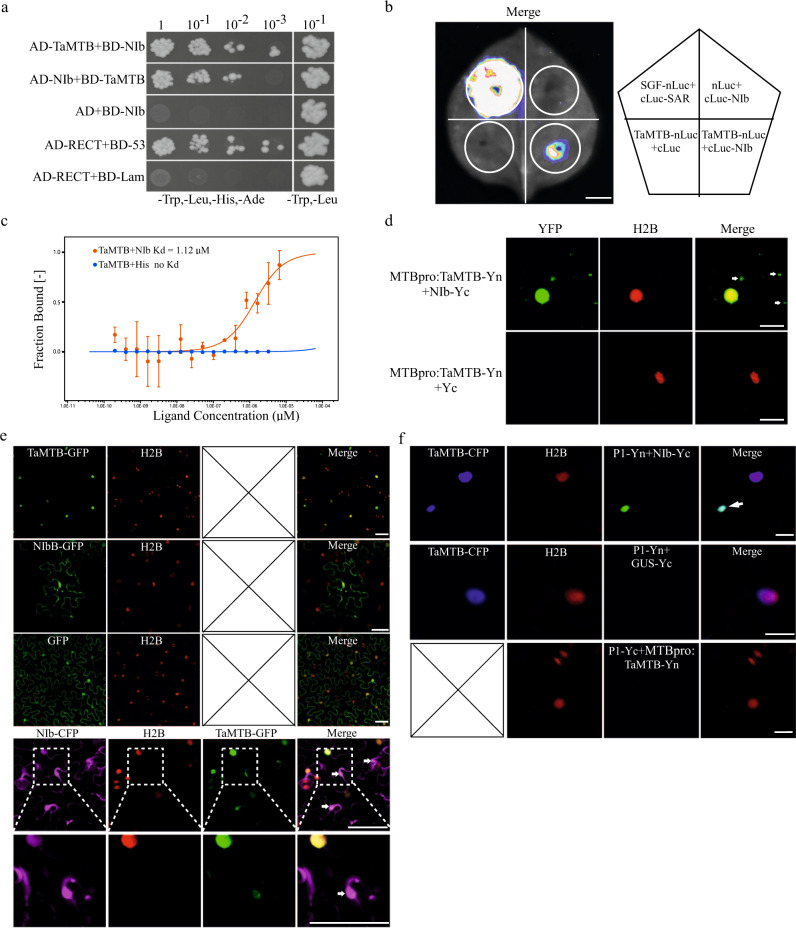
*TaMTB* is recruited into the cytoplasmic aggregates by interacting with NIb. **a** Yeast-two hybrid assay for interaction between TaMTB and NIb. Yeast co-transformed with AD-RECT + BD −53 served as a positive control **b** Interaction of TaMTB with NIb detected by split luciferase complementation imaging (LCI) assay on *N. benthamiana* leaves. The leaf areas infiltrated with Agrobacterium cultures expressing SGF-nLuc+cLuc-SAR were used as a positive control, TaMTB-nLuc+cLuc and nLuc+cLuc-NIb were used as negative controls, respectively. Luciferase activity was captured using a low-light cooled CCD imaging apparatus at 3dpi. **c** In vitro interaction of TaMTB-His (from *E. coli*) with NIb detected by microscale thermophoresis (MST). The mixture with TaMTB protein+His was used as a negative control. The solid curve is the fit of the data points to the standard Kd-Fit function. Each binding assay was repeated three times independently (*n* = 3), and error bars represent SD. Data in (**c**) are represented as means ± SD. Kd, dissociation constant. **d** BiFC assay was used to evaluate TaMTB-NIb interaction. TaMTB and NIb were fused to the N (Yn) and C-terminal (Yc) fragments of yellow fluorescent protein (YFP). TaMTB-Yn was driven by its native promoter (MTBpro). The TaMTB-NIb interaction led to the reconstituted fluorescence-competent structure and restoration of yellow fluorescence (green). Nuclei of tobacco leaf epidermal cells are indicated by the expression of H2B-RFP transgene (red). Arrow indicates the cytoplasmic aggregates. Bars, 20 μm. **e** Sub-cellular localization of TaMTB-GFP, NIb-GFP and co-localization of TaMTB-GFP with NIb-CFP (purple) in the leaf cells of H2B-RFP transgenic *N. benthamiana* by confocal microscopy at 48 hpi. localization of 35 S:GFP was used as control. Arrow indicates the cytoplasmic aggregates where TaMTB and NIb coincide. The corresponding region in the white box was magnified below it. Bars, 50 μm. **f** Co-localization of P1-Yn + NIb-Yc or Gus-Yc with TaMTB-CFP (blue). P1-Yn+Gus-Yc+TaMTB-CFP was used as negative control. And TaMTB-Yc+P1-Yn was used to evaluate TaMTB-P1 interaction. Arrow indicates the cytoplasmic aggregates where TaMTB and NIb-P1 complex coincide. Confocal images were performed at 48 hpi and three times each experiment was repeated independently with similar results. Bars, 20 μm. Source data are provided as a Source Data file.

Viruses rely on a complex interaction network with host factors to complete infection. To understand the molecular mechanism involved in the regulation of WYMV infection by TaMTB, we performed a yeast two-hybrid (Y2H) assay using TaMTB as a bait to screen a WYMV cDNA library. Sequencing of positive clones showed that the WYMV NIb accounted for the largest proportion of positive interactors. This interaction was then confirmed in yeast cells via an Y2H assay (Fig. 3a) and further verified by a split luciferase complementation imaging (LCI) assay in the *N. benthamiana* cells co-expressed with NIb-cLUC and TaMTB-nLUC (Fig. 3b). To further examine whether TaMTB physically interacts with NIb, we performed a microscale thermophoresis (MST) assay in vitro using the purified TaMTB and NIb proteins from *E. Coli* (Supplementary Fig. 5). A dissociation constant (Kd) of 1.12 μM was measured for the binding form of TaMTB+NIb, suggesting that TaMTB indeed interacts with NIb in vitro (Fig. 3c).

To further investigate the influence on MTB protein by their interaction, we performed a biomolecular fluorescence complementation (BiFC) assay. To avoid the effects of 35 S promoter-driven gene overexpression, *TaMTB* fused to N-terminus of YFP was expressed under the control of its native promoter (MTBpro:TaMTB-Yn). The BiFC result in transgenic *N. benthamiana* leaves (express H2B-RFP, a nuclear marker) showed that the interaction between TaMTB and NIb occurs in both the nucleus and cytoplasmic aggregates (Fig. 3d). We also repeated the assay using three segments of TaMTB, in which MTB^1–298,299aa^ contains no known domain, MTB^300–505,506*aa*^ contains a Med15 domain and MTB^507–802,803*aa*^ contains a MT-A70 domain. Both the results of BiFC assay and co-IP assay showed that only MTB^1–298,299aa^ interacted with NIb in vivo (Supplementary Fig. 6). To further verify the effect of NIb on TaMTB, we performed subcellular localization analysis in tobacco. TaMTB protein fused with GFP tag (TaMTB-GFP) was localized in the nucleus, and NIb-GFP was localized in both the nucleus and cytoplasm (Fig. 3e). Interestingly, when TaMTB-GFP co-expressed with NIb-CFP (NIb protein fused with CFP), the two proteins not only colocalized in the nucleus but also in some cytoplasmic aggregates (Fig. 3e). Moreover, we constructed TaMTB-CFP, P1-Yn and NIb-Yc recombinant plasmids and co-expressed them in H2B tobacco leaves. As shown in Fig. 3f, some TaMTB-CFP was re-distributed to the P1-Yn-NIb-Yc labeled punctate structures in the cytoplasm, while TaMTB seemed to have no interaction with WYMV P1 (Fig. 3f, Supplementary Fig. 7). Therefore, these data demonstrated that TaMTB is recruited into the cytoplasmic protein aggregates (that consist of P1 and NIb) by its interaction with NIb protein.

### TaMTB is a member of m^6^A writers in wheat

**Fig. 4:**
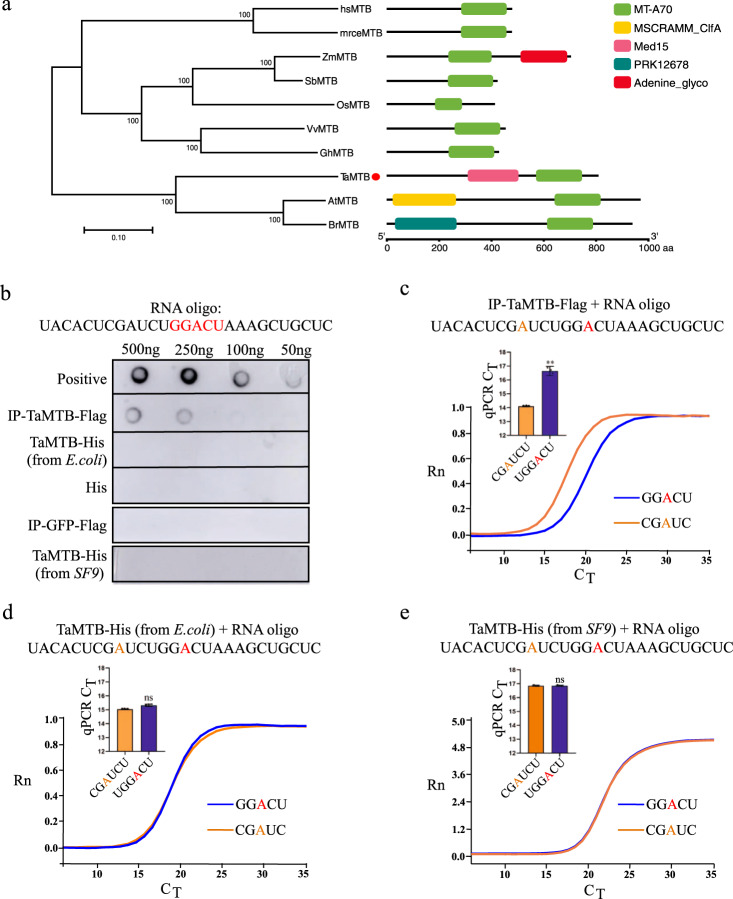
TaMTb is a member of writers of m^6^A in wheat. **a** Phylogenetic and conserved domain analysis of TaMTB from difference species. The MTB protein sequences were obtained from *Homo sapiens* (hs), *Mus musculus* (mrce), *Zea mays* (Zm), *Sorghum bicolor* (Sb), *Oryza sativa* (Os), *Vitis vinifera* (Vv), *Gossypium hirsutum* (Gh), *T. aestivum* (Ta), *Arabidopsis thaliana* (At), *Brassica rapa* (Br). TaMTB is highlighted in the phylogenetic tree using a red circle. The phylogenetic tree was constructed by the neighbor-joining method in MEGA 7.0 software, with bootstrap values of 1000. The percentage on phylogenetic tree nodes indicated that the associated taxa clustered together in the bootstrap test. The branch lengths represent evolutionary distances, which are calculated using p-distance method. **b** In vitro methylation assay of TaMTB by detection with dot blot. The part marked in red represents the m^6^A motif. The RNA oligo with m^6^A modification (listed in Supplementary Data 3) was used as positive control. IP-GFP-Flag and His protein was used as negative controls. IP-TaMTB-Flag and IP-GFP-Flag were purified from 35 S:TaMTB-Flag and 35 S:GFP-Flag transfected wheat protoplasts. TaMTB-His (*E. coli*) was purified from *E. coli*. TaMTB-His (*Sf9*) was purified from *Sf9 cells*. **c, d, e** Real-time fluorescence amplification curves and bar plot of the threshold cycle (C_T_) of qPCR showing SELECT results for detecting the m^6^A site in RNA oligo with IP-TaMTB-Flag, TaMTB-His (*E. coli*) and TaMTB-His (*Sf9*) treatment. Rn is the raw fluorescence for the associated well normalized to the fluorescence of the passive reference dye (ROX). Values are means ± SD (two-sided *t* test, n  =  3, **(c)** *P*  <  0.0001). **P  <  0.01. ns, not significant. The red or yellow marked A represent the base that are possible to be m^6^A methylated. Source data are provided as a Source Data file.

To reaffirm the identity and function of TaMTB protein, we undertook a bioinformatical analysis by constructing a phylogenetic tree with TaMTB sequence compared to its homologs from other species. The result showed that TaMTB protein shares a close relationship with *Arabidopsis* MTB (AtMTB) and *Brachypodium distachyon* MTB (BrMTB), all containing a MT-A70 conserved domain (Fig. 4a) that has been reported to be responsible for methylation activity^46^. To verify the methyltransferase activity of TaMTB, IP-TaMTB-Flag was purified from 35 S:TaMTB-Flag transfected wheat protoplasts and Two different TaMTB-His were purified from *E. coli* and *Sf9* cells, respectively (Supplementary Fig. 5, 7). His-tag protein purified from *E. Coli* and GFP-Flag protein from wheat mesophyll protoplasts were used as negative controls (Supplementary Fig. 8). Then, an in vitro methylation assay combined with Dot blot assay was used to detect the m^6^A modification of RNA oligos. Only TaMTB-Flag, and not TaMTB-His(*E. coli*)/TaMTB-His(*Sf9*)/His/GFP-Flag could transfer the methyl group of SAM to the adenine in the synthesized RNA oligos (Fig. 4b). From SELECT analysis, an elongation- and ligation-based qPCR quantification method for detection of single m^6^A locus at single-base resolution^47^. The result confirmed that m^6^A modification could be detected again on the IP-TaMTB-Flag treated RNA oligo but not detected on TaMTB-His(*E. coli*) and TaMTB-His(*Sf9*) treated RNA oligos (Fig. 4c–e). These results indicated that IP-TaMTB-Flag recombinant protein has m^6^A activity so probably acts as a writer of m^6^A. Nevertheless, further study is required to understand why TaMTB-His(*E. coli*) and TaMTB-His(*Sf9*) protein had no activity.

### TaMTB positively regulates m^6^A level of WYMV RNA1 by binding to it

**Fig. 5:**
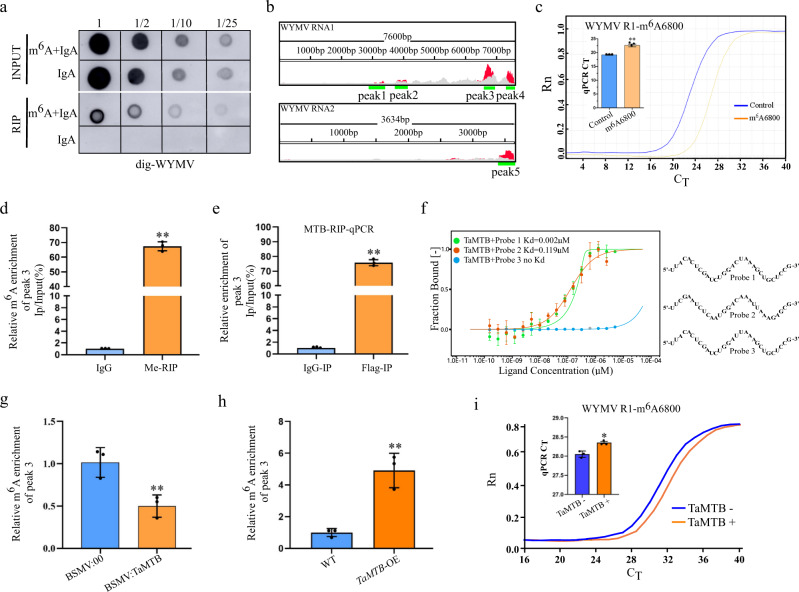
TaMTB positively regulates m^6^A level of WYMV R1. **a** RNA immunoprecipitation (RIP) of WYMV RNAs with a specific anti-m^6^A antibody. Total RNA extracted from WYMV-infected wheat plants was incubated with anti-m^6^A plus IgA or IgA alone. Dilutions of the immunoprecipitated RNAs were blotted on Hybond-N^+^ membrane and detected with Dig-WYMV. **b** m^6^A peaks in WYMV RNA1 and RNA2. The detail peaks and viral gene annotation are shown in Supplementary Table 2. Top numbers show the full length of the analyzed RNA segments, bp, base-pair. Green color lines are the m^6^A region. Red, IP peak. Gray, Input peak. **c** Real-time fluorescence amplification curves and bar plot of the threshold cycle (C_T_) of qPCR showing SELECT result for detecting the m^6^A site in peak3 of RNA 1. Rn is the raw fluorescence for the associated well normalized to the fluorescence of the passive reference dye (ROX). Values are means ± SD (two-sided *t* test, *n*  =  3, *P*  <  0.0001). **P  <  0.01. **d** m^6^A-IP-qPCR assay of peak3 in WYMV-infected wheat. Total RNA extracted from WYMV-infected wheat plants incubated with anti-m^6^A or IgG (negative control) for IP. Values are means ± SD (two-sided *t* test, *n*  =  3, *P*  <  0.0001). **P  <  0.01. **e** RIP-qPCR assay revealing the binding of MTB to peak3 region. The protein-RNA complexes were extracted from WYMV-infected *TaMTB-Flag*-OE wheat plants and subjected to immunoprecipitation with anti-Flag or mouse IgG (negative control). Values are means ± SD (two-sided *t* test, *n*  =  3, *P*  <  0.0001). **f** In vitro interaction of TaMTB-His(*E. coli*) with probes 1-3 detected by MST experiment. TaMTB protein+ probe 3 was used as a negative control. Each binding assay was repeated three times independently, and error bars represent SD. Data in (**f**) are represented as means ± SD. m^6^A-IP-qPCR assay of peak3 in WYMV-infected BSMV:00 / BSMV:TaMTB **(g)** or wild type (WT) / *TaMTB*-OE **(h)** plants. BSMV:00 and WT were used as negative controls, respectively. Values are means ± SD (two-sided *t* test, *n*  =  3, **(g)** *P*  = 0.0074, **(h)** 0.0021). **i** SELECT analysis for detecting m^6^A6800 site in RNA 1 w**i**th or without TaMTB treatment. Values are means ± SD (two-sided *t* test, *n*  =  3, *P*  <  0.0001). Source data are provided as a Source Data file.

We next tested for the presence of m^6^A modification in WYMV genomic RNAs. Total RNA was isolated from WYMV-infected wheat plants and an RNA immunoprecipitation assay (RIP) using the anti-m^6^A antibody was performed to immunoprecipitate the m^6^A-modified RNAs. Hybridization of RIP products with the Dig-WYMV probes through Dot blot assay clearly detected the presence of the WYMV genomic RNAs (Fig. 5a), indicating that m^6^A was present in WYMV genomic RNAs. To further identify the specific m^6^A, a m^6^A-IP-seq analysis of WYMV-infected wheat samples was performed (PRJNA694346). When the clean reads from m^6^A sequencing data were aligned to the WYMV reference genomic RNA, we found m^6^A methylation occurring both in RNA1 and RNA2 with four obvious peaks in the coding region of RNA1 and one peak in the 3’ terminal of RNA2 (Fig. 5b, Supplementary Table 2). SELECT analysis was then used to validate the specific m^6^A sites in WYMV genomic RNAs. Six potential m^6^A sites from these five peaks were predicted using SRAMP (Supplementary Table 3) and chosen to be analyzed by SELECT, only one specific m^6^A sites from peak 3 (6800th adenosine residues) eventually identified (Fig. 5c, Supplementary Fig. 9). Meanwhile, the m^6^A modification of peak3 was also confirmed by m^6^A-IP-qPCR analysis (Fig. 5d). Moreover, RNA immunoprecipitation (RIP)-qPCR assay indicated that TaMTB directly binds to the WYMV RNA1 in vivo (Fig. 5e). To further confirm this, we selected 30 base sequences of peak3 region containing the GGA^6800^CA motif as RNA probe 2 and used it for a MicroScale Thermophoresis **(**MST) experiment to investigate the binding of TaMTB to the peak3 region, using RNA probe 1 and 3 as the positive and negative control^29^. Although the Kd value 0.119 μM of TaMTB-His(*E. coli*) + probe 2 was higher than 0.002 μM of the positive control, this still suggested the binding of TaMTB-RNA probe 2 in vitro (Fig. 5f). Subsequently, we assessed the m^6^A level of peak3 region through m^6^A-IP-qPCR in the WYMV-infected BSMV:00 and BSMV: TaMTB (*TaMTB* - silenced) wheat plants. The m^6^A level of peak3 was significantly down-regulated in BSMV:TaMTB plants in comparison to BSMV: 00 plants (Fig. 5g). In contrast, the peak3 in WYMV-infected *TaMTB*-OE wheat plants displayed higher m^6^A levels than the control of WYMV-infected WT plants (Fig. 5h). Furthermore, SELECT assays in two parallel total RNA samples with or without IP-TaMTB-Flag treatments were conducted and confirmed that TaMTB is responsible for the m^6^A addition at the GGA^6800^CA motif of WYMV RNA1 (Fig. 5i). In summary, data suggested that TaMTB directly binds WYMV RNA1 through recognizing the GGA^6800^CA motif in WYMV RNA1 and positively regulates the m^6^A level of WYMV RNA1.

### Inactivation of the m^6^A site of WYMV RNA1 destabilizes viral RNA and slows viral infection

**Fig. 6:**
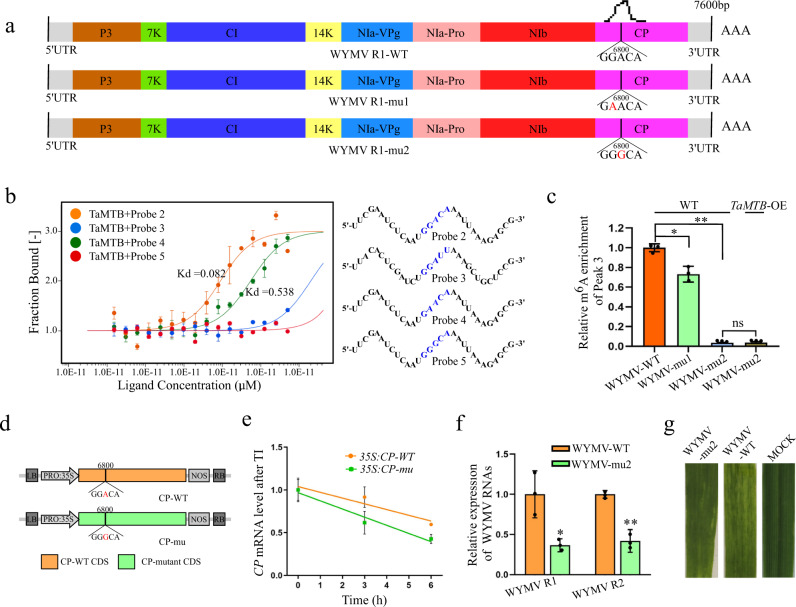
Mutations in GGACA motif of WYMV RNA1 slows the viral infection. **a** Schematic diagram of two WYMV R1 mutations. The mutated bases are marked in red. The different colored boxes represent the coding regions of different proteins. The black peak represents m^6^A peak. **b** Binding affinity of TaMTB-His(*E. coli*) with probes 2-5 (right) detected by MST experiment. The mixture with TaMTB protein+ probe 3 was used as a negative control. The solid curve is the fit of the data points to the standard Kd-Fit function. Each binding assay was repeated three times independently, and bars represent SD. Data in (**b**) are represented as means ± SD. Kd, dissociation constant. The blue sequence represents the m^6^A motif. **c** m^6^A-IP-qPCR assay to detect the m^6^A level of peak 3 in WYMV-WT or WYMV-mu1/2 infected WT wheat plants and WYMV-mu2-infected *TaMTB*-OE plants. Values are means ± SD (two-sided *t* test, *n*  =  3, *P*  = 0.0116, *P*  <  0.0001). **P  <  0.01 *P  <  0.05. ns, no significant. **d** Schematic diagram of the CP mutation used for mRNA stability assay. The wild type (WT) or mutated (mu) coding region of *WYMV CP* was cloned into the expression vector driven by the CaMV 35 S promoter. **e** The mRNA lifetime of *WYMV CP*. TI: transcription inhibition. Data are represented as means ± SD for 3 biological replicates (two-sided *t* test, *P*  = 0.0153, 0.0085). **f** The accumulation of WYMV RNA1 and RNA2 in WYMV-WT-infected and WYMV-mu2-infected wheat plants were determined by qRT-PCR using *CP* and *P2* gene-specific primers. Values are means ± SD (two-sided *t* test, *n*  =  3, *P* =  0.0192, *P*  =  0.0021). **g** Phenotypes in the fourth leaves of the plants inoculated with phosphate buffered saline (MOCK), WYMV-WT or WYMV-mu2, respectively. Source data are provided as a Source Data file.

To further investigate whether the GGA^6800^CA motif is significant for m^6^A modification of WYMV RNA1, two WYMV infectious cDNA clone mutants were constructed: WYMV-mu1(mutated G^6799^ to A) and WYMV-mu2 (mutated A^6800^ to G) (Fig. 6a). MST assay was then used to evaluate the binding affinity of TaMTB-His(*E. coli*) with WYMV R1-WT or WYMV R1-mu1/2. 30 base sequences (as shown in Fig. 6b) in peak 3 region of WYMV R1-mu1/2 was selected as RNA probes 4 and 5. Probes 2 and 3 (Fig. 5f) were the respective positive and negative control. The Kd values of TaMTB-probes 2/4 were 0.082 μM and 0.538 μM respectively but no Kd value for TaMTB plus probe 5 (Fig. 6b), which suggested that the mutation of GGACA motif leads to a decreased or even none binding affinity of TaMTB to RNA probe. Moreover, the m^6^A level of peak 3 in WYMV-mu1/2 infected wheat plants were also down-regulated than WYMV-WT infected plants and overexpression of *TaMTB* did not restore the m^6^A level in WYMV-mu2 (Fig. 6c), indicating that the GGACA motif is essential for m^6^A modification of WYMV R1 by TaMTB. Furthermore, we constructed a m^6^A-inactivation mutation form of *WYMV CP* (*CP-mu*) and *CP-WT*, then respectively transient expressed in the wheat protoplasts (Fig. 6d). The m^6^A modification has been reported to be associated with mRNA degradation and stabilization^48^, thus we further performed transcription inhibition assays using actinomycin D to measure the lifetime of *CP-mu* and *CP-WT*. The results showed that *CP-mu* transcripts degraded more rapidly compared to *CP-WT* (Fig. 6e). Subsequently, we detected WYMV accumulation both in WYMV-mu2 or WYMV-WT inoculated seedlings through qRT-PCR; inferring that the accumulation of WYMV-mu2 RNAs were significantly lower than WYMV-WT RNAs (Fig. 6f, g). Consistently, the mosaic symptoms in WYMV-mu-inoculated plants were also milder than in WYMV-WT-infected plants (Fig. 6h). Taken together, our results demonstrated that GGACA motif of WYMV R1 is essential for its m^6^A modification and the abolishment of m^6^A destabilizes the WYMV RNA 1 and consequently reduces the viral infection.

### A natural *TaMTB* variation causes changes in wheat resistance to WYMV

**Fig. 7:**
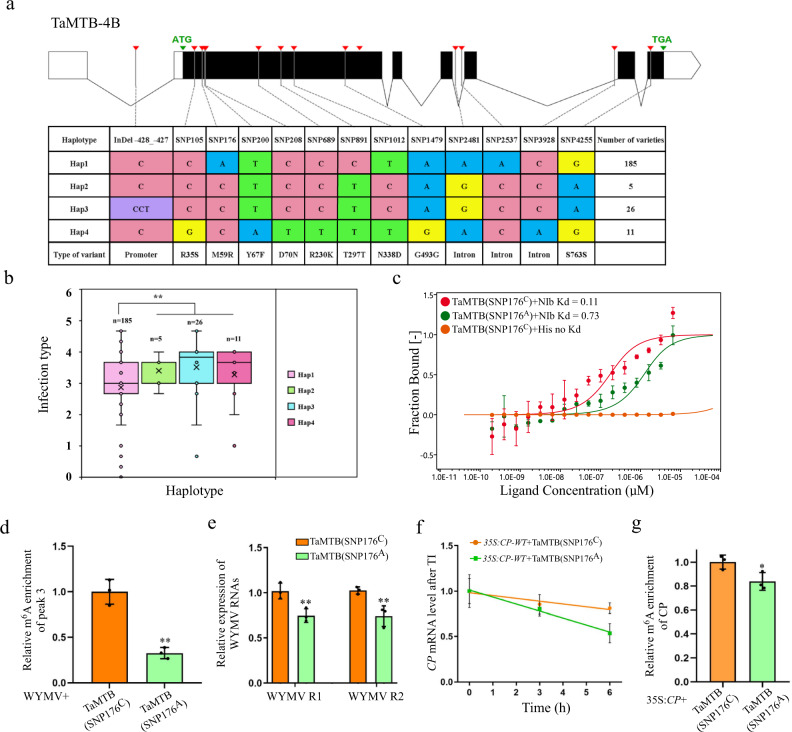
Mutation of SNP176^A/C^ in TaMTB promotes viral infection. **a** Gene structure display and DNA polymorphisms of *TaMTB-B1*. The black box represents the CDS region, the blank space represents the UTR region. Different colors represent different types of bases. **b** Comparison of WYMV resistance between wheat varieties carrying Hap 1-4 genotypes. The infection type is derived from the mean value of all replicates. *n* represent the number of wheat accessions with the corresponding haplotype. Statistics: for both datasets, two-sided *t* test was performed, *P* =  0.0014, **P  <  0.01. **c** Binding affinity of TaMTB(SNP176^A^) or TaMTB(SNP176^C^) with NIb detected by microscale thermophoresis (MST) experiment. The mixture with TaMTB(SNP176^C^) + His was used as a negative control. The solid curve is the fit of the data points to the standard Kd-Fit function. Each binding assay was repeated three times independently, and bars represent SD. Data points are represented as means ± SD. **d** m^6^A-IP-qPCR assay to detect the m^6^A level of peak 3 in WYMV-infected wheat plants which transient expressed TaMTB(SNP176^A^) or TaMTB(SNP176^C^). Values are means ± SD (two-sided *t* test, *n*  =  3, *P* =  0.01) **P  <  0.01. **e** The accumulation of WYMV RNA1 and RNA2 in WYMV-infected wheat plants which transient expressed TaMTB(SNP176^A^) or TaMTB(SNP176^C^) were determined by qRT-PCR using *CP* and *P2* gene-specific primers. Values are means ± SD (two-sided *t* test, *n*  =  3, *P* =  0.0335, 0.0248) *P  <  0.05. **f** The mRNA lifetimes of *WYMV CP*. Data are represented as means ± SD for 3 biological replicates (two-sided *t* test, *P*  = 0.0172). TI: transcription inhibition. **g** m^6^A-IP-qPCR assay to detect the m^6^A level of *CP* mRNA in *N. benthamiana* leaves co-expressed with 35 S:CP-GFP + TaMTB(SNP176A) or 35 S:CP-GFP + TaMTB(SNP176C). Values are means ± SD (two-sided *t* test, *n*  =  3, *P* =  0.0419) *P  <  0.05. Source data are provided as a Source Data file.

To further investigate whether genetic variation in TaMTB is associated with differences in resistance to WYMV among wheat varieties, we performed sequence analysis of *TaMTB-B1* which indicated that 4 haplotypes were fromed in 243 varieties, and *TaMTB-B1_*Hap1 exhibited significantly the strongest WYMV resistance among four haplotypes (Fig. 7a, b). In addition, using the single nucleotide polymorphism 176, SNP176^A/C^ (a non-synonymous nucleotide polymorphism with an amino acid change from methionine to arginine at position 59) as the polymorphism marker, two alleles *TaMTB*^*S*^ and *TaMTB*^*R*^ can be distinguished (Fig. 7a, b). Additionally, an independent panel containing 119 wheat varieties further revealed a significant association of the variant site SNP176^A/C^ with WYMV resistance (Supplementary Fig. 10). Moreover, sequencing of the *TaMTB-B1* in Xianyangdasui/Xinmai208 also identified the SNP176^A/C^ (Supplementary Fig. 11). We, therefore, deduced that this variation of SNP176^A/C^ may be responsible for the observed differences in phenotypic resistance. Following these results, we cloned *TaMTB (SNP176*^*A*^*)* and mutated 176A to 176 C to create *TaMTB(SNP176*^*C*^*)*, then both were expressed in *E. Coli* and two proteins TaMTB(SNP176^A^) and TaMTB(SNP176^C^) were finally purified (Supplementary Fig. 12). MST assay was performed to investigate the binding affinity between NIb and SNP176^A^ or SNP176^C^, the binding affinity of NIb-TaMTB (SNP176^C^) (Kd=0.11 μM) was 6.6-fold higher than that of NIb-TaMTB (SNP176^A^) (Kd=0.73 μM) (Fig. 7c). Furthermore, we also expressed TaMTB (SNP176^A^) or TaMTB (SNP176^C^) transiently in the WYMV-infected *N. benthamiana* leaves and confirmed it by Western blot after 48 hpi (Supplementary Fig. 13a). The m^6^A level of peak3 in these leaves was significantly higher in leaves expressing TaMTB (SNP176^C^) than in those expressing TaMTB (SNP176^A^) (Fig. 7d). Correspondingly, the accumulation of WYMV was much higher in the leaves expressing TaMTB (SNP176^C^) (Fig. 7e). The lifetime of *WYMV CP* transcript was also longer when co-expressed with TaMTB (SNP176^C^) than when co-expressed with TaMTB (SNP176^A^) (Fig. 7f, Supplementary Fig. 13b). The m^6^A level of *CP* in 35 S:*CP*-wt-GFP + TaMTB(SNP176^A^) was slightly lower than that of 35 S:*CP*-wt-GFP + TaMTB(SNP176^C^). Moreover, in a SELECT analysis, the methyltransferase activity of of IP-TaMTB-Flag (SNP176^A^) was also lower than that of IP-TaMTB-Flag (SNP176^C^) (Supplementary Fig. 14). Therefore, we speculated that the variation A/C of TaMTB may also be associated with its methyltransferase activity (Fig. 7g). In summary, these results suggested that *TaMTB* (SNP176^C^) allele was significantly associated with WYMV susceptibility and it increases both the binding affinity to NIb protein and its methyltransferase activity to enhance the m^6^A level of WYMV RNA1 thus stabilizing the WYMV RNA1 so as to promote viral infection.

## Discussion

Plant genes which promote pathogen infection and suppress host immune responses are referred to as susceptibility (*S*) genes^49^. In crop plants, loss-of-function mutations in *S* genes could result in durable broad‐spectrum resistance (BSR) which is genetically recessive and non-pathogen-specific^4^. Therefore, generation of new crop varieties with BSR by modifying *S* genes via CRISPR/Cas9 has been developed. However, *S* genes also often function in plant growth and development, and thereby disrupting S genes may produce some pleiotropic effects like plant growth defects and crop yield penalty^49,50^. Pathogen resistance and grain yield are the two major targets of crops breeding^51^, so balancing plant immunity with growth is critical for the application of S genes. Thus, elite rice varieties containing a recessive resistance mutated allele *ROD1* (SNP1^A^) exhibit higher pathogen resistance but without any obvious yield penalty^7^, suggesting that *ROD1* is a good candidate for pathogen resistance engineering in rice.

In this study, we located the *TaMTB* gene by combination of GWAS and QTL, and functionally verified it as an *S* gene for WYMV (Fig. 1). We have used 243 Chinese wheat accessions to carry out a genome-wide association (GWAS) study to identify WYMV resistance loci with the Wheat 660 K SNP array (Fig. 1a) that demonstrated a potential in wheat genotyping and marker-assisted selection^52^. Then an interval of 595–610 Mb covering 129 annotated genes has been colocalized from GWAS combined with QTL mapping (Fig. 1. b, c). From 129 gene transcriptional profiles, *TaMTB-B1* was recognized as the most significant gene associated with WYMV resistance (Fig. 1). Therefore, this gene was cloned and overexpressed in a wild-type wheat ‘Fielder’ to investigate its function. *TaMTB* overexpression enhanced the susceptibility to WYMV infection (Fig. 2). Although we were unable to create a viable loss-of-function mutant, knock-down of *TaMTB* expression decreased viral infection (Fig. 3). These results indicate that TaMTB acts as a host positive regulator to promote WYMV infection in wheat.

Identification of the methyltransferase is essential for understanding the functions of m^6^A modification in any organism. In humans, two methyltransferases, METTL3 and METTL14 participate in m^6^A modification as ‘writers^29^. MTB is the homolog of human METTL14 in plants, which has also been shown to be a part of m^6^A methyltransferase complex^23^, but there is no published research focusing solely on MTB and its role in m^6^A methylation. Růžička and colleagues found that m^6^A level was reduced to 50% in *MTB-RNAi Arabidopsis* lines^23^. METTL14 may have lost catalytic activity, but it has an allosteric role to support METTL3 active site, playing a critical role in substrate RNA recognition^30^. Another study showed that METTL14 purified from insect cells had very weak methyltransferase activity^29^. Similarly, our study also found that the TaMTB purified from *E. coli* or *Sf9* cells had no methyltransferase activity, but a reasonable high activity was measured in TaMTB purified from wheat cells (Fig. 4). It is possible that some endogenous METTL homologs, which were co-purified with the MTB protein complexes from wheat cells but not from bacterial cells, integrated with and activated TaMTB. It is also possible that the methyltransferase activity of TaMTB needs to be activated by some unusual post translation modifications that only occur in wheat cells. Although several ‘WRITERs’ have been identified in Arabidopsis, ‘WRITERs’ from other plant species have rarely been reported. Here, TaMTB is identified as a m^6^A ‘WRITER’ protein in wheat.

As the canonical m^6^A methyltransferase complex is predominantly nuclear^29^, m^6^A modification studied previously has only been with viruses that replicate in the nucleus. However, recent studies have shown that m^6^A modification is also present in viruses that replicate in the cytoplasm, including the mammalian hepatitis C virus (HCV)^40^, Zika virus (ZIKV) and Vesicular Stomatitis Virus (VSV)^41^ and the plant viruses alfalfa mosaic virus (AMV)^18^, rice black-stripe dwarf virus (RBSDV) and rice stripe virus (RSV)^43^. In this study, we have also demonstrated that the genome of WYMV (an RNA virus that replicates in the cytoplasmic) is modified by m^6^A and we further identified an adenosine 6800 in the CDS region of WYMV RNA1 that is modified to m^6^A (Fig. 5a–d), suggesting that m^6^A modification may also be common amongst viruses that replicate in the cytoplasmic. TaMTB directly binds to the peak 3 region containing A6800 of WYMV in vivo and in vitro, and positively regulated the m^6^A level of peak3 in wheat plants (Fig. 5e–i). Moreover, mutation of the m^6^A modification site in *CP* gene reduced the stability of *CP* transcripts (Fig. 6d, e) and in an infectious clone of WYMV with this mutation, virus accumulation was decreased compared to the wild-type virus (Fig. 6f), consistent with the result from plants where *TaMTB* was silenced. In contrast, m^6^A modification in 3’ epsilon stem loop of HBV transcript reduced their stability^36^. This difference regulation of RNA stability may be dependent on the m^6^A distribution in the nexus of the local sequence contexts within transcripts, which results in the binding of diverse m^6^A readers that carry the distinct and even opposite molecular functions^53^. However, it is still unclear in plants how these cytoplasmic replicative viruses gain m^6^A.

The Viral replication center (VRC) is a functional unit that consists of viral replication proteins, a viral RNA template, and diverse host proteins. Without these important host factors the VRC will not form or function normally thus suppressing viral infection^54^. For example, glyceraldehyde-3-phosphate dehydrogenase (GAPDH) is recruited by tomato bushy stunt virus (TBSV) p92 to VRC and GADPH-downregulation inhibits TBSV replication and infection^55^. eEF1A, interacts with p33 and p92, is also a component of the TBSV VRC and eIF1A mutants inhibit the assembly of the VRC and synthesis of negative-sense RNA^56^. In this study, we found that a host factor, TaMTB interacted with WYMV NIb (Fig. 3a–c). As in Arabidopsis and strawberry^26,57^, TaMTB was localized in the nucleus (Fig. 3d). When co-expressed with NIb, some TaMTB was translocated from the nucleus to cytoplasm punctate aggregates (Fig. 3d, e). A previous studies had indicated that WYMV NIb could interact with WYMV P1 protein and colocalized with WYMV P2 induced replication-associated inclusion bodies^58^. We here demonstrated that some TaMTB re-distributes to P1-Yn+NIb-Yc induced cytoplasm aggregates (Fig. 3f). It is known that localization of METTL3/14 (MTA/B plants homolog protein) can be regulated by numerous interacting RNA-binding proteins^32^. METTL3 and METTL14 are recruited to viral RNA replication sites by interacting with the 3D RNA-dependent RNA polymerase of Enterovirus 71^59^. NIb is the RNA-dependent RNA polymerase of WYMV^8^ and our study suggests that TaMTB is a host factor of WYMV, and we speculate that it may be recruited into the WYMV VRCs by NIb protein.

We also found two natural alleles *TaMTB* (SNP176^A^) and *TaMTB* (SNP176^C^) in 243 wheat varieties, showing a different binding affinity to WYMV NIb with the higher affinity allele *TaMTB* (SNP176^C^) having an increased m^6^A level to stabilize WYMV RNA 1 in infected cells (Fig. 7c–g). Our data also indicated that the natural variation of *TaMTB* (SNP176^A/C^) does not alter crop growth and yield but modifies the host resistance to WYMV, suggesting that *TaMTB* may represent an elite target for wheat molecular breeding to achieve both high yield and high resistance to WYMV disease.

**Fig. 8:**
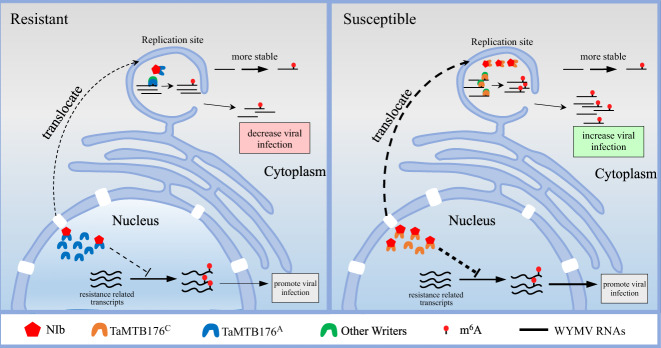
Model for N6-methyladenosine RNA modification promotes viral genomic RNA stability and infection. During WYMV infection, NIb protein recruits TaMTB into the WYMV replicate sites then directly binds to WYMV RNA1 for m^6^A modification. This m^6^A modification stabilizes the WYMV RNA1 for a longer lifetime to promote WYMV accumulation. In addition, TaMTB (SNP176^C^) protein has a higher binding affinity for NIb compared to TaMTB (SNP176^A^) enhancing recruitment into VCR and mediating m6A modification to stabilize WYMV RNA1 to enhance viral infection. Therefore, wheat varieties containing TaMTB (SNP176^C^) allele are more susceptible to WYMV than those with the TaMTB (SNP176^A^) allele. The NIb-TaMTB interaction may also suppress the m^6^A modification levels of resistance related genes to further promote WYMV infection.

Taken together, we propose a model illustrating a form of viral pathogenesis in plants in which the host methyltransferase TaMTB is translocated to cytoplasmic VRCs by interacting with WYMV NIb protein, activating m^6^A modification on WYMV RNA1 to stabilize viral RNA so as to promote viral infection. The natural variation of TaMTB176^A/C^ alters the binding affinity with WYMV NIb thus leading to different WYMV resistance in wheat. Our previous study had shown that the host m^6^A modification levels were significantly altered after WYMV infection and we, therefore, speculate that the interaction of NIb with TaMTB may also promote WYMV infection by interfering the m^6^A modification levels of resistance related genes (Fig. 8).

## Methods

### Plant materials and growth conditions

A total of 362 wheat accessions, composed of an association panel containing 243 current varieties^60,61^ and 119 historical varieties^62,63^, were planted in wheat yellow mosaic virus (WYMV) nursery field in Xiping (N33.40°, E113.3°) in 2016-2017 cropping seasons with one replicate and in the 2017-2018 and 2018-2019 cropping seasons with two replicates. An F_8_ RIL (recombinant inbred lines) population composed of 138 lines, derived from the cross between Xianyangdasui (resistant to WYMV) and Xinmai 208 (susceptible to WYMV), was also planted in the same field with two replicates in 2017-2018, 2018-2019, 2019-2020 and 2020-2021 cropping seasons. Xinmai 18 was planted as highly susceptible control every 10 lines. All the tested materials grew well. Disease severity was investigated at the jointing stage when Xinmai 18 reached the level 3 infection type (IT)^11^.

Wheat seedlings of cv. Yangmai 158 (YM158) and cv. Fielder were grown in a glasshouse at 25 °C. WYMV-inoculated wheat seedlings were grown in a climate chamber at 10 ± 2 °C with a 16 h light/8 h dark photoperiod. For field tests, YM158, Fielder and *TaMTB*-OE transgenic wheat lines were grown in a WYMV disease nursery in Yangzhou, Jiangsu, China, from 2020 to 2022.

### Evaluation of WYMV resistance infection type

For all test accessions, WYMV infection types (ITs) were investigated three times every 10 days from mid-February to mid-March each year. Based on the maximum severity of WYMV occurrence, the ITs were recorded to evaluate the WYMV resistance of each test accession^11^, where

IT = 0: No yellow symptom presents in the test plants;

IT = 1: Less than 50% of plants with yellow symptom;

IT = 2: More than 50% of plants with yellow symptom but no dwarfing occurs;

IT = 3: All plants with yellow symptom but no dwarfing;

IT = 4: All plants with yellow symptom and plants slightly dwarfed;

IT = 5: All plants with yellow symptom and the plants seriously dwarfed.

The WYMV resistance of these plants which belongs to different ITs were also confirmed by qRT-PCR (Supplementary Fig. 15).

### Genotyping and genome-wide association study

All 243 wheat accessions were genotyped using the Wheat 660 K SNP array^60,61^. After quality control, only SNPs with minor allele frequency (MAF) > 0.05 and missing data <20% in the association panel were kept for GWAS analysis using PLINK software^62,64^. GWAS analysis was implemented using GAPIT packages in R software based on the mixed linear model (PCA + K)^65^. The population structure of the association panel was constructed by ten subpopulations^66^. The threshold of the significant *P*-value was set as 1.0E-3 for all environments.

### Linkage mapping and quantitative trait locus

The RIL population XX (Xianyangdasui/Xinmai208) (XX-RIL) was genotyped using the Wheat 55 K SNP array. A total of 15,062 markers were polymorphic between two parents, and then a 10 Mb interval was used for haplotype analysis. In each haplotype, tagSNPs were randomly selected, and the representative 8301 markers were used to construct a genetic linkage map. After the bin function operation, 1739 (including 83 heterozygous) markers were used for quantitative trait locus (QTL) mapping using IciMapping V4.1. nnTwoOpt and SAD were used to complete linkage algorithm and standard measurement^67^. The threshold of LOD score was set as 2.5 to indicate the existence of a QTL.

### Bulked segregant RNA-seq in bi-parents RIL population

Three resistant pools (IT < 1) and three susceptible pools (IT > 4) of the XX-RIL population were used for bulked segregant RNA-Seq. Each WYMV-resistant or WYMV-susceptible pools were composed of an equivalent mixture of leaves from 10 lines of the RIL population. Sampled leaves were rapidly frozen in liquid nitrogen and stored at −80 °C for bulked segregant transcriptome using RNA-seq (BSR-seq).

### Plasmid construction

The full length of WYMV NIb, P1, CP and TaMTB-4B were respectively cloned from a WYMV infectious clone and Yangmai158 cDNA. The mutated forms of CP-mu, TaMTB (SNP176C), WYMV R1-mu1 and WYMV R1-mu 2 were constructed using the overlap PCR. The full-length WYMV RNA1-mu 1/2 were inserted into the *BamHI/SacI*-digested plasmid pCB301 vector, resulting in pCB301-SP6-R1-mu1 and pCB301-SP6-R1-mu2. Full-length NIb and TaMTB were inserted into pGBKT7/pGADT7 vector via ClonExpress MultiS One Step Cloning Kit (Vazyme, China). pET32a-NIb-His, pET32a-TaMTB-His and pET32a- TaMTB(SNP176A/C)-His were constructed by inserting the NIb and TaMTB CDS into pET-32a(+) via One Step Cloning. TaMTB and NIb CDS were respectively cloned into pCambia1300-n/cLUC to obtain TaMTB-nLUC and NIb-cLUC. p35S:NIb-Yc, p35S:P1-Yn/c, p35S:TaMTB-Yn, and recombinant fluorescent plasmid for transient expression were all constructed using the Gateway technology (Invitrogen). The first PCR used the primer pairs which listed in Supplementary Data 3. The second PCR was performed using primers attB1 and attB2 and the amplified products of the first PCR as a template. These amplified products were introduced into pDONR207 by the BP reaction and then these fragments were further transferred into the destination vectors, respectively. The upstream promoter region of TaMTB was cloned into the p35S:TaMTB-Yn (pGTQL1211) plasmids instead of the 35S promoter to obtain MTBpro:TaMTB-Yn. All primers used in this study were listed in Supplementary Data 3.

### qRT-PCR analysis

Total RNAs were extracted from the wheat plants using the plant RNA extraction kit (Magen, R4165-02) then reverse transcribed into cDNAs by the Rever Tra Ace qPCR RT Master Mix (TOYOBO, FSQ-201) and used as templates for PCR amplification. Quantitative real time (qRT)-PCR analysis was performed using an ABI Q5 Sequence Detection System (Applied Biosystems, Foster City, CA, USA) with an AceQ qPCR SYBR Green Master Mix (Vazyme). At least three biological triplicates were used for each assay. The *T. aestivum* cell division cycle (CDC) gene (XM_020313450 [https://www.ncbi.nlm.nih.gov/nuccore/2158015422]) was used as internal reference genes for analysis to calculate the fold changes in gene expression. The fold changes were calculated using the 2^-ΔΔC(t)^ method^68^. All gene-specific primers for qRT-PCR are shown in Supplementary Data 3.

### BSMV-based VIGS

300 bp fragment of TaMTB sequence contains two restriction enzyme sites *PacI* and *NotI* was inserted into pCa-γ vector to obtain γ-TaMTB. BSMV RNA α, β and γ or γ-TaMTB were linearized to plasmid DNAs and then transcribing into RNAs in vitro using the Ribo MAX^TM^ Large Scale RNA Production Systems-T7 and the Ribo m7G Cap Analog (both by Promega). The resulting transcripts were mixed then diluted with 7 μl inoculation buffer (0.06 M potassium phosphate, 0.1 M glycine, 1% bentonite, 1% sodium pyrophosphate decahydrate, 1% celite, pH 8.5) in a ratio 1:1:1:7. BSMV transcripts mixture was rub-inoculated to the leaf 2 (from the bottom) of a wheat seedling at the three-leaf stage. The inoculated seedlings were grown inside a dark growth chamber at 25 °C and high humidity for 24 h, and then under a16 h:8 h, light: dark photoperiod.

### Inoculation of wheat seedlings with WYMV RNAs

WYMV RNA1 and RNA2 were linearized with *Spe*I restriction digestion and transcribed in vitro using the Promega Ribo MAX^TM^ Large Scale RNA Production Systems-Sp6 and the Ribo m7G Cap Analog following the manufacturer’s instructions. Transcripts WYMV RNA1 and RNA2 were mixed in a 1:1 ratio and diluted two-fold with RNase-free H_2_O. Next, 2 μl of mixed WYMV RNAs were diluted with 8 μl of inoculation buffer (0.06 M potassium phosphate, 0.1 M glycine, 1% bentonite, 1% sodium pyrophosphate decahydrate, 1% celite, pH 8.5) and used to rub-inoculate the second leaf of a wheat seedling at the three-leaf stage. The inoculated seedlings were grown at 8 °C and 80% humidity in a constant temperature and humidity incubator.

### Wheat transformation

The chosen fragment of TaMTB CDS were cloned and inserted into the *BamH* I and *Sal* I sites followed by the pCAMV35S promoter in the expression vector pCAMV35S:00 to produce pCAMV35S:TaMTB-Flag. The vector pAHC20 containing a selective marker gene (herbicide resistance gene) was co-transformed with pCAMV35S:TaVTC2 into immature wheat embryos by particle bombardment^8^.

### Co-immunoprecipitation

*N. benthamiana* leaves were co-infiltrated with GV3101 cultures carrying the pCAMV35S:NIb-GFP plus GV3101 cultures carrying pCAMV35S:MTB^1–298,299aa^-Flag or pCAMV35S:MTB^300–505,506*aa*^-Flag or pCAMV35S:MTB^507–802,803*aa*^-Flag, respectively. 60 h post infiltration, the leaves were collected and ground in liquid nitrogen. Total proteins were extracted from 2 g of ground powder in 4 ml of IP buffer (50 mM Tris-HCl pH 7.5, 150 mM NaCl, 5 mM MgCl2, 2 mM EDTA, 0.5% Triton X-100, 5% glycerol, 5 mM DTT, 1 mM PMSF) supplemented with Protease Inhibitor Cocktail Tablets (1 tablet/50 ml buffer; Roche, 4693116001). The extract was centrifuged three times at 20,000 × g for 10 min each time at 4 °C and the supernatant was collected. Twenty-five microliters of anti-GFP magnetic beads (Abbkine Scientific Co., Ltd., California, USA, KTI2024) was added to the supernatant and incubated at 4 °C for 2 h with rotation. After incubation, the beads were washed three times with IP buffer at 4 °C. Finally, 50 μl of 2 × SDS loading buffer were added to the sample for western blot analyses.

### Western blot

Proteins was collected and mixed individually with SDS loading buffer boiled for 8 min. For immunoblot, proteins were separated in 10% SDS-PAGE gels through electrophoresis, and then transferred to NC membranes. The blots were probed with anti-CP (1:2000, prepared by Huaan Bio, Hangzhou, Zhejiang, China, stored in our lab), anti-Flag and anti-GFP (1:5000, TransGen Biotech, Beijing, China, Cat. No. HT201-01 and HT801-01), followed by an HRP-conjugated secondary mouse antibody (1:5000, Abbkine Scientific Co., Ltd., California, USA, Cat. No. A21010). The detection signals were developed using an ECL reagent as instructed (Thermo Scientific, Hudson, NH, USA), and visualized using a Bio-Rad ChemiDoc Touch imaging system (Bio-Rad, Hercules, CA, USA).

### Yeast two-hybrid assay

Yeast two-hybrid (Y2H) assays were performed following the method described in the Takara protocol handbook (Takara Bio Inc, Japan). In brief, Yeast cells (strain Y2H Gold) carrying the co-transformed plasmids (AD-TaMTB/BD-TaMTB, AD-NIb/BD-NIb) were plated onto a low-stringency selective medium lacking tryptophan and leucine (SD/-Trp-Leu) to confirm the transformation and then plated onto a high-stringency selective medium lacking tryptophan, leucine, histidine, and adenine (SD/-Trp-Leu-His-Ade) to analyze the interaction. Transformants carrying empty pGADT7 (AD) vector were used as negative controls, and that concurrently carrying pGBKT7-53 and pGADT7-RECT vectors was used as the positive control.

### LCI assay

TaMTB-nLUC and NIb-cLUC were separately transformed into *Agrobacterium* strain GV3101. The agrobacteria were cultured at 28 °C for 18 h in YEP liquid medium containing 50 μg/mL kanamycin, gentamycin and rifampicin. GV3101 containing expression plasmids were centrifugated at 8000 × *g* for 30 s. Collected strains carried TaMTB-nLUC and NIb-cLUC were separately resuspended in buffer (10 mM MES, pH 5.6, 10 mM MgCl2, 200 mM acetosyringone), and then diluted to an OD600 = 0.6 with infiltration medium before leaf infiltration. Next, two GV3101 strains were mixed in a 1:1 volume ratio and infiltrated into *N. benthamiana* leaves then culture at 25 °C for 72 h. After that, the leaves were incubated with 1 mM luciferin dissolved in ddH2O supplemented with 0.01% Triton X-100 at room temperature for 5 min, and then observed under a chemiluminescence imaging system (Tanon). Empty vectors expressing cLUC or nLUC were co-transformed as the negative controls. SGF-nLUC and SAR-cLUC were co-transformed as the positive controls^69^.

### MicroScale thermophoresis assay

The affinity of the purified TaMTB for NIb or RNA probes (listed in Supplementary Data 3) was investigated using Monolith NT.115 (NanoTemper Technologies). MicroScale thermophoresis (MST) labelling of TaMTB was conducted in PBS solution containing a Monolith NT protein labelling kit RED according to the manufacturer’s instructions (NanoTemper Technologies). Samples containing labelled protein and ligands with different concentration gradients were then loaded into NanoTemper hydrophilic-treated capillaries. The resulting samples were analyzed by the manufacturer using NanoTemper analytical software to estimate their equilibrium dissociation constant Kd values.

### BiFC and sub-cellular localization

*Agrobacterium* strain GV3101 containing expression plasmids were centrifuged for 30 s at 8000 rpm, resuspended in buffer (10 mM MES, pH 5.6, 10 mM MgCl_2_, 200 mM acetosyringone), and then diluted to an OD600 of 0.8. For BiFC, GV3101 strains containing p35S:NIb-nYFP or p35S:TaMTB-cYFP were resuspended and adjusted to an OD600 with infiltration medium before leaf infiltration. Next, two GV3101 strains were mixed in a 1:1 ratio and infiltrated into *N. benthamiana* leaves then culture at 25 °C for 72 h. The expression of fluorescent proteins was examined at 48 h post agroinfiltration under a Leica TCS SP8 confocal laser scanning microscope (Leica Microsystems, Heidelberg, Germany).

### In vitro **methylation assay**

Recombinant plasmid Pet32a-TaMTB-His was transformed into the *Escherichia coli* strain Rosetta-gami (DE3). The TaMTB-His was purified followed the manufacturer’s instructions of Ni-NTA 6FF Sefinose (TM) Resin Kit (BBI C600332). 35 S:TaMTB-Flag recombinant plasmid was transfected into wheat protoplasts using a PEG-mediated transformation method (Bio-Rad, Hercules, CA, USA). The transfected mesophyll protoplasts were cultured in W5 solution at 23 °C for 18 h in the dark. Then TaMTB-Flag protein was purified by Anti-flag ® M2 magnetic beads (Sigma). To express TaMTB-His in Sf9 cells, PCR production of TaMTB CDS with C-terminal His_6_ was inserted into pFast-bac1 between *BamH I* and *Hind III*. Recombinant baculoviruses were prepared using the Bac-to-Bac Baculovirus Expression System and transfected into *Sf9* cells using lipofectamine (Invitrogen). The *Sf9* cells density was kept at 2 × 106 cells/ml. The cells were cultured in a shaking incubator at 27 °C for 72 h and collected by centrifugation at 500 × *g* for 5 min. Then, TaMTB-His was purified as described above. In vitro methylation assay was performed as previously described^29^. In brief, a standard 50 μl of reaction mixture for methylation assay was composed of 0.15 nmol RNA probe (synthesized in Sangon, China), 0.15 nmol protein (TaMTB-His or TaMTB-Flag), 0.8 mM SAM (A4377, Sigma), 80 mM KCl, 1.5 mM MgCl2, 0.2 UμL^−1^ RNasin, 10 mM DTT, 4% glycerol, and 15 mM HEPES (pH 7.9). Prior to the reaction, the RNA probes were annealed with a program of (i) 90 °C for 3 min and (ii) −2 °C/cycle for 40 cycles within 30 min. The reaction was incubated at 16 °C for 12 h. RNA was purified through phenol/chloroform extraction followed by ethanol precipitation. The sequence of the RNA probe (listed in Supplementary Data 3) was referenced by Liu et al.^29^.

### Dot blot

In short, mRNA was divided into different concentration gradients then spotted to Hybond-N + membrane, followed by UV crosslinking at UV 254 nm with 0.12 J/cm^2^. After blocking in TBST buffer containing 5% non-fat milk for 1 h, the membrane was incubated with 1:2000 diluted anti-m^6^A antibody (Cat. No. 202 003, Synaptic Systems, Göttingen, Germany) 2 h at 4 °C. The membrane was washed with TBST three times and then incubated with HRP conjugated secondary antibody (1:5000, Abbkine Scientific Co., Ltd., California, USA, Cat. No. A21010) for 1 h. And visualized by using Immobilon^TM^ Western HPR Substrate Luminol Regeant (Merck Millipore).

### SELECT assay

A volume of 17 μl pre-mixed solution containing 50 ng of mRNAs, 40 nM Up-Primer, 40 nM Down-Primer, and 5 μM dNTP, 1× CutSmart buffer, 20 mM Tris-HAc, pH 7.9, 50 mM KAc, 10 mM MgAc_2_, and 100 μgml-1 BSA. Pre-mixed solution was incubated at a temperature gradient: 90 °C for 1 min, 80 °C for 1 min, 70 °C for 1 min, 60 °C for 1 min, 50 °C for 1 min, and then 40 °C for 6 min. Then, 0.01 U Bst 2.0 DNA polymerase (NEB, M0537S), 0.5 U SplintR ligase (NEB, M0375S), and 10 nmol ATP were added to the mixture to a final volume of 20 μL, followed by incubating at 40 °C for 20 min and denatured at 80 °C for 20 min. Finally, the mixture was used to qRT-PCR analysis with SELECT universal primer pair listed in Supplementary Data 3.

### m^6^A RIP of viral genomic RNAs

Extracted total RNAs (200 μg) from WYMV-infected wheat plants were incubated in a final volume of 500 μL mixture containing 1×IP buffer [50 mM Tris·HCl, 150 mM NaCl, 0.5% (vol/vol) Nonidet P-40], 200 U RNasin and 10 μg m^6^A antibody (Cat. No. 202 003, Synaptic Systems, Göttingen, Germany) in rotation for 2 h at 4 °C. Next, 200 μL of recombinant protein A beads in 1× IP buffer were added into 500 μL mixture and additionally rotation for 2 h at 4 °C. Then, beads were collected and washed four times with 1× IP buffer followed by incubating with elution competition buffer [50 mM Tris·HCl, 150 mM NaCl, 0.5% (vol/vol) Nonidet P-40, 200 U RNasin and 6.7 mM m^6^A] for 1 h with vigorous shaking to elute m^6^A enriched RNAs. And m^6^A enriched RNAs were purified through phenol/chloroform extraction followed by ethanol precipitation. the same reactions without specific m^6^A antibody were used as negative control. Eluted RNAs was diluted into different concentration gradients and directly blotted onto Hybond-N + membrane and hybridized with Dig-WYMV probes which listed in Supplementary Data 3.

### m^6^A-IP-qPCR

A total of 2.5 μg purified mRNAs were fragmented into ~300 nucleotide-long fragments by an incubation at 94 °C for 30 s in the RNA fragmentation buffer (10 mM Tris-HCl, pH 7.0, and 10 mM ZnCl_2_), followed by the addition of 50 mM EDTA to terminate the reaction. Fragmented mRNAs were purified through phenol/chloroform extraction followed by ethanol precipitation. Specifically, 10 percent of fragmented mRNAs were saved as input sample and the rest of RNAs were used for Immunoprecipitation (IP) which was performed the same as the m^6^A RIP of viral genomic RNAs. the input mRNAs and IP mRNAs were reverse transcribed with Rever Tra Ace qPCR RT Master Mix (TOYOBO). The relative mRNA enrichment was measured using qRT-PCR and normalized to the input level. Primers used are listed in Supplementary Data 3.

### MTB-RNA immunoprecipitation (RIP)-qPCR assay

Leaves of WYMV-infected *TaMTB*-OE wheat plants were fixed with 1% formaldehyde under a vacuum for 30 min on ice. The fixation was terminated by the addition of 150 mM glycine, followed by incubation on ice for 5 min. The fixed leaves were grinded with N_2_ and homogenized in 5 mL of lysis buffer (50 mM HEPES, pH 7.5, 2 mM EDTA, 150 mM KCl, 0.5% NP-40 (v/v), 2 mM EDTA, 0.5 mM DTT, 1× cocktail protease inhibitor (Roche), and 200 U/mL RNase). After incubation in rotation for 2 h at 4 °C, the mixture was centrifugated at 15,000 *g* for 20 min at 4 °C. Then, 500 μL of the supernatant were collected as the input control, and the rest was used to IP with 10 μg anti-Flag monoclonal antibody (TransGen Biotech, Beijing, China, HT201-01) or 10 μg rabbit lgG (invitrogen, USA, 10500C) at 4 °C overnight. After that, 60 μl of Dynabeads Protein-A (Cat. No. AG-A1003, Synaptic Systems, Göttingen, Germany) were added to the mixture and incubated at 4 °C for 2 h followed by washing three times with PBS buffer. the RNA-protein mix was catalyzed by proteinase K (Takara, 9034) at 55 °C for 1 h then the IP RNAs and input RNAs were reverse transcribed for qRT-PCR assay and normalized to the input level. the lgG immunoprecipitated RNAs were used as negative control. Primers used are listed in Supplementary Data 3.

### mRNA stability assay

Briefly, p35S:CP-GFP and 35S:CP-mu-GFP were transfected into wheat protoplast and incubated for 18 h at room temperature. After these, 20 μg/mL actinomycin D (Sigma, A4262) dissolved in ddH_2_O were added into transfected wheat protoplasts. After culture for 30 min, wheat protoplasts were taken and considered as time 0 controls, and subsequent samples were harvested every 3 h in triplicate. The mRNA levels of genes were subsequently examined by qRT-PCR assay.

For mRNA stability assay in *N. benthamiana* leaves, recombinant expression plasmids were infiltrated into the *N. benthamiana* leaves by Agrobacterium. After 36 h of incubation, the infiltrated leaves were injected with 20 μg/mL actinomycin D (Sigma, A4262). Then mRNA stability was assayed as described above.

### Reporting summary

Further information on research design is available in the Nature Research Reporting Summary linked to this article.

## Supplementary information

Supplementary Information

Description of Additional Supplementary Files

Supplementary Data 1

Supplementary Data 2

Supplementary Data 3

Reporting Summary

## Data Availability

Data supporting the findings of this work are available within the paper and its Supplementary Information files. The raw m^6^A-seq plus RNA-seq data are available in the NCBI database under accession code PRJNA694346. Source data are provided with this paper.

## Source data

Source Data
