# Supplementary material for: N6-methyladenosine RNA modification promotes viral genomic RNA stability and infection
Source: Nat Commun. 2022 Nov 2;13:6576. doi: 10.1038/s41467-022-34362-x (PMC9629889; doi:10.1038/s41467-022-34362-x)
Supplement: Supplementary file 1 — Supplementary Information [file 41467_2022_34362_MOESM1_ESM.pdf]

N^6-methyladenosine RNA modification promotes viral genomic RNA stability and infection

Zhang *et al.*

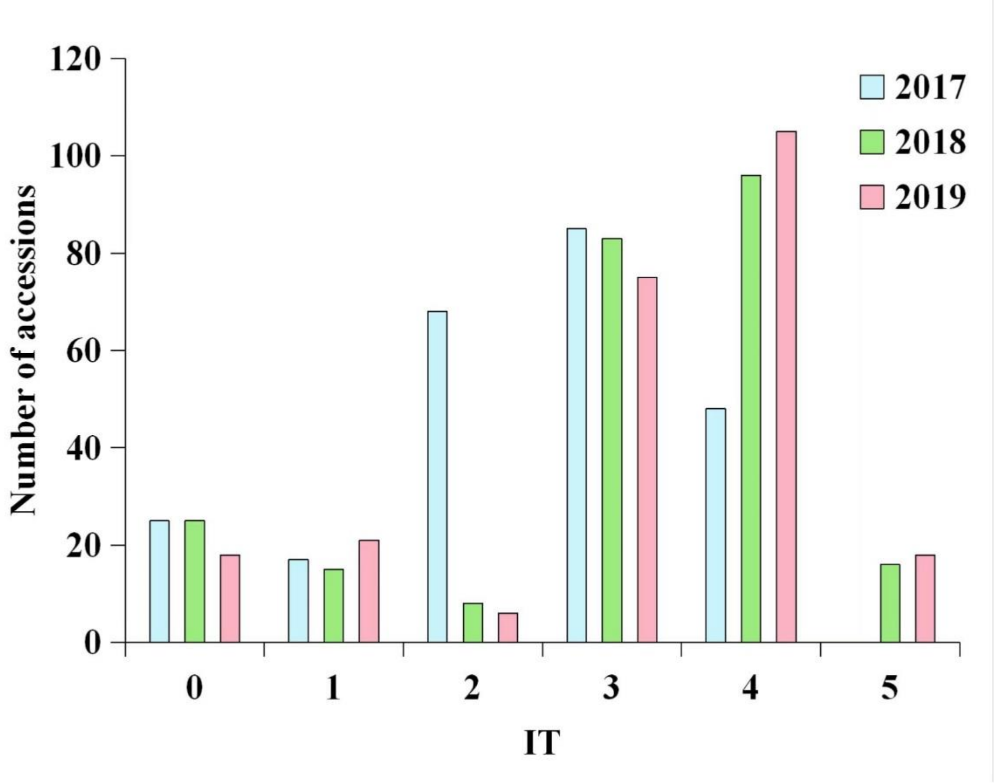

Supplementary Figure 1. Phenotypic distribution and analysis of associated populations for three consecutive years. IT, infection types.

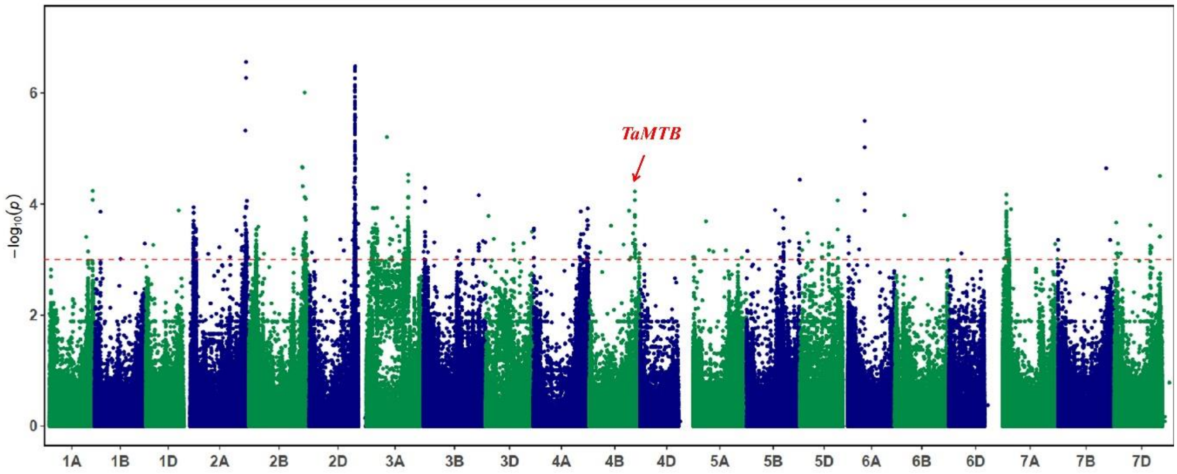

Supplementary Figure 2. Manhattan plots of the mixed linear model for WYMV. Negative $\log_{10}$-transformed *P* values from a genome-wide scan are plotted against position on each of 21 chromosomes. Red horizontal dashed line indicates the genome-wide significance threshold.

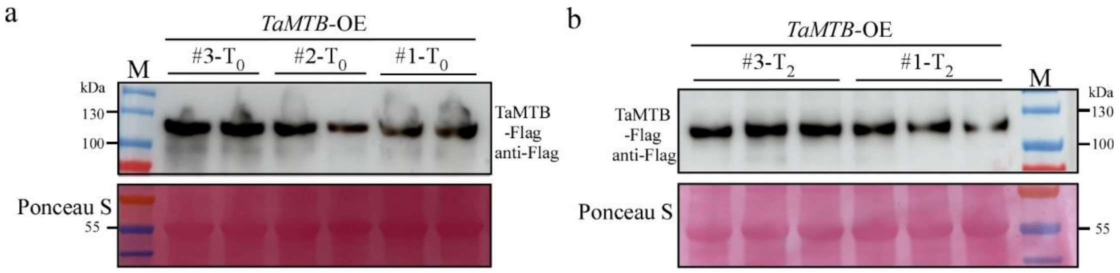

Supplementary Figure 3. Screening positive transgenic plants of *TaMTB*-OE. Detection of TaMTB expression in T0 **(a)** or T2 **(b)** generation *TaMTB*-OE plants by western blot using a Flag-specific antibody. #1,2, 3 was two independent transgenic lines of *TaMTB*-OE. Three times each experiment was repeated independently with similar results . Source data are provided as a Source Data file.

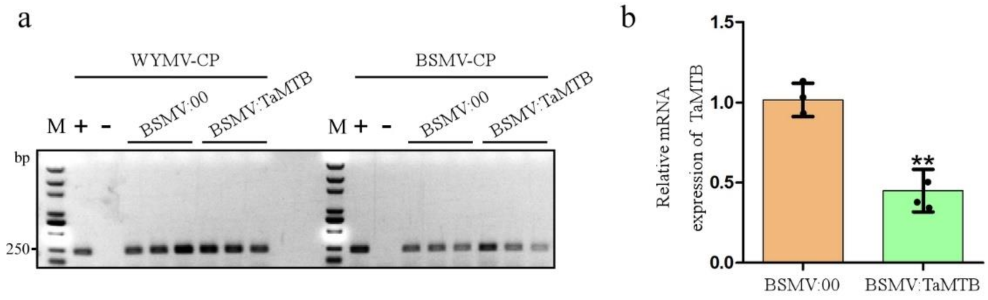

Supplementary Figure 4. RT-PCR and qRT-PCR analysis. a Detection of BSMV and WYMV infection in BSMV:00+WYMV or BSMV:TaMTB+WYMV inoculated plants by RT-PCR using BSMV CP and WYMV CP specific primers. **b** Relative expression levels of TaMTB in plants co-infected with WYMV + BSMV: TaMTB or WYMV + BSMV: 00. Total RNA from a BSMV:00 + WYMV co-infected wheat plants was normalized to 1. Values are means ± SD (two-sided t test, n = 3, $P = 0.0032$) **$P < 0.01$. Source data are provided as a Source Data file.

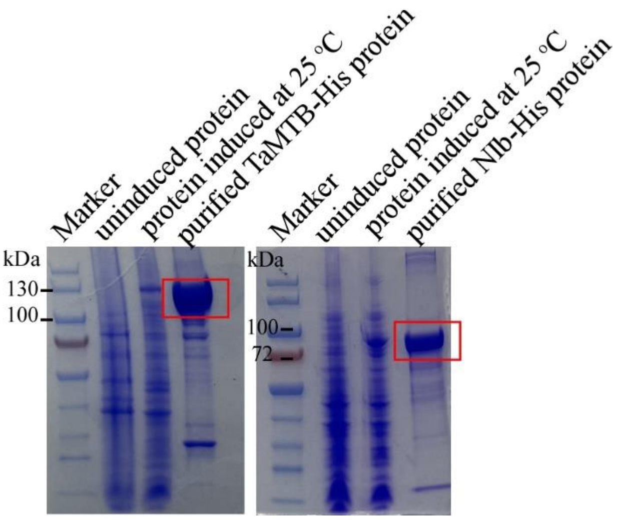

Supplementary Figure 5. Coomassie blue staining of purified proteins. The red box shows the target protein. Three times each experiment was repeated independently with similar results. Source data are provided as a Source Data file.

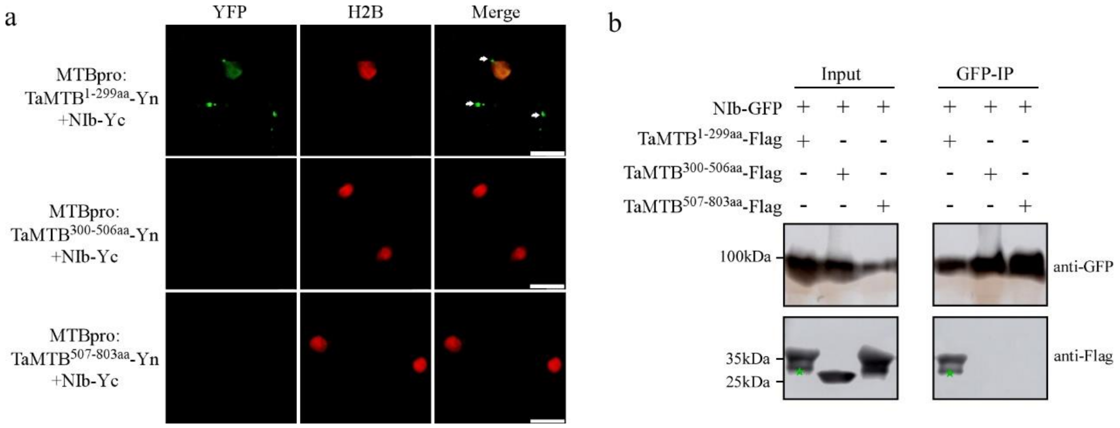

Supplementary Figure 6. **TaMTB$^{1-299aa}$ is the key region for its interaction with NIb. a** BiFC assay was used to evaluate the key region of TaMTB for its interaction with NIb. TaMTB$^{1-299aa}$/TaMTB$^{300-506aa}$ TaMTB$^{507-803aa}$ and NIb were fused to the N (Yn) and C-terminal (Yc) fragments of yellow fluorescent protein (YFP). TaMTB$^{1-299aa}$/TaMTB$^{300-506aa}$ TaMTB$^{507-803aa}$-Yn was driven by its native promoter (MTBpro). The TaMTB^{x-xaa}-NIb interaction led to the reconstituted fluorescence-competent structure and restoration of yellow fluorescence (green) Confocal imaging was performed at 48 hpi and three times each experiment was repeated independently with similar results. Nuclei of tobacco leaf epidermal cells are indicated by the expression of H2B-RFP transgene (red). Arrow indicates the cytoplasmic aggregates. Bars, 20 μm. **b** Co-immunoprecipitation analysis of the interactions of TaMTB$^{1-299aa}$/TaMTB$^{300-506aa}$/TaMTB$^{507-803aa}$ with NIb. Green star, unknown protein. Results are representative of three independent experiments. Source data are provided as a Source Data file.

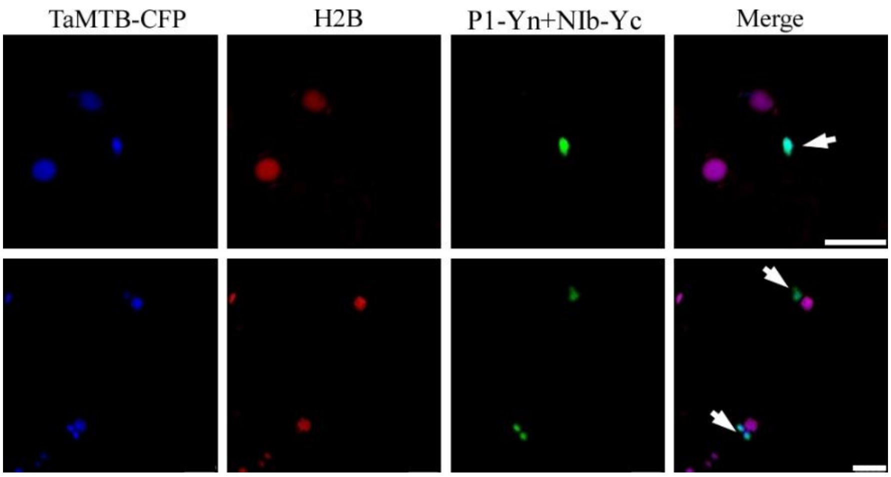

Supplementary Figure 7. TaMTB co-localizes with P1-NIb protein aggregates in cytoplasm. NIb-P1 complex were showed in green and TaMTB-CFP was showed in blue. Nuclei of tobacco leaf epidermal cells are indicated by the expression of H2B-RFP transgene (red). Confocal imaging was performed at 48hpi and three times each experiment was repeated independently with similar results. Arrow indicates the cytoplasmic aggregates. Bars, 25 μm.

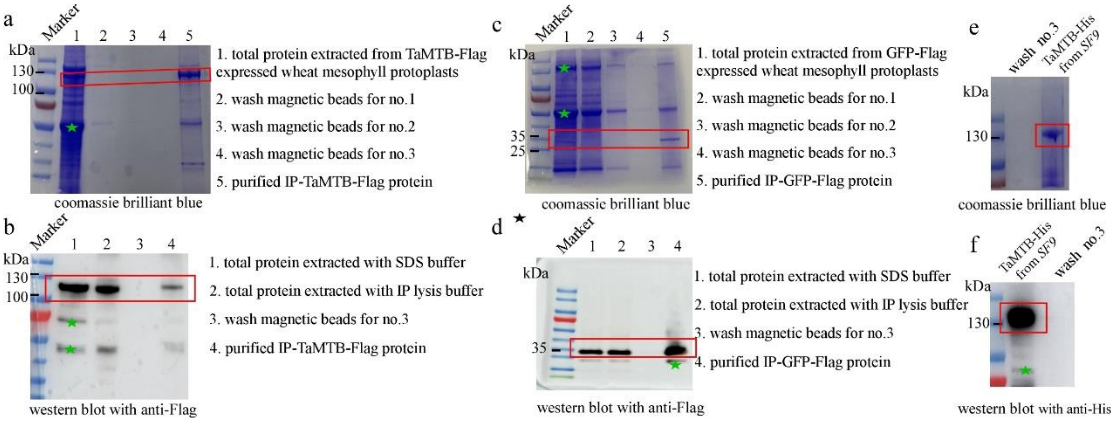

Supplementary Figure 8. Coomassie blue staining and Western blot assay of purified IP-TaMTB-Flag (a, b), IP-GFP-Flag (c, d) and TaMTB-His (from *SF9*) (e, f). IP-TaMTB-Flag and IP-GFP-Flag purified from wheat protoplasts, TaMTB-His purified from *SF9* cells. The red box shows the target protein. Green star, unknown protein. Three times each experiment was repeated independently with similar results. Source data are provided as a Source Data file.

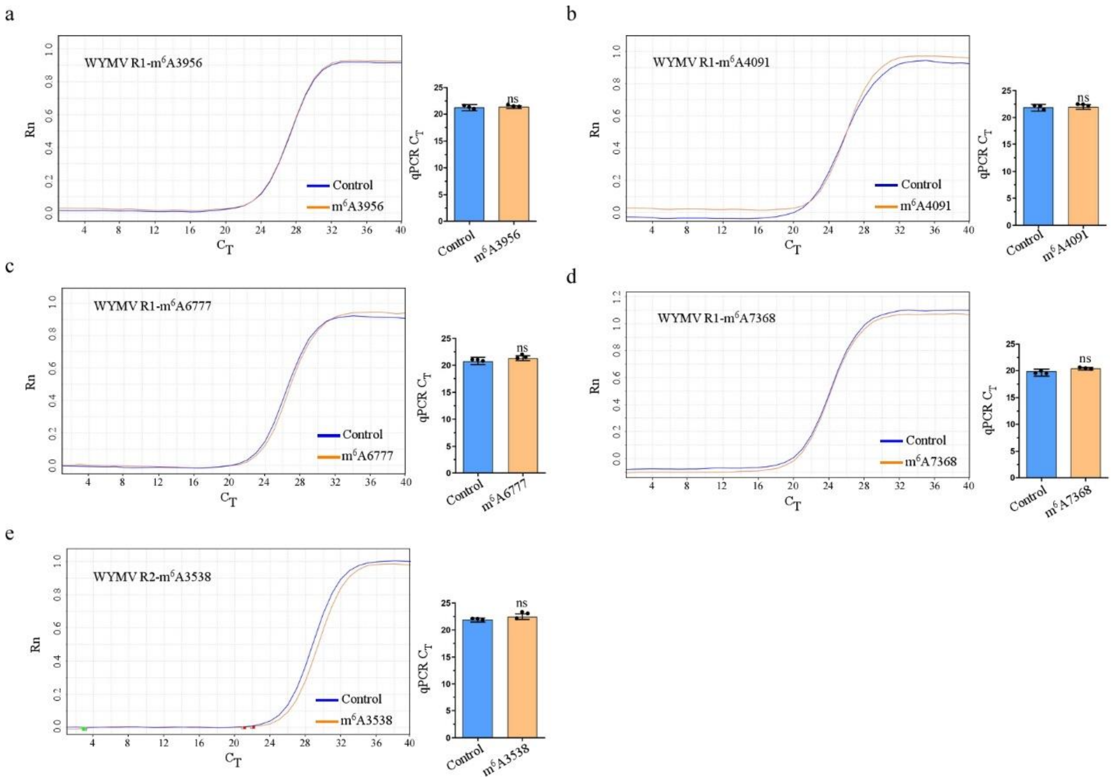

Supplementary Figure 9. SELECT analysis for detecting a series of single m^{6}A sits. Real-time fluorescence amplification curves and bar plot of the threshold cycle (C_T) of qPCR showing SELECT result for detecting a series m^{6}A sites in WYMV RNAs. Rn is the raw fluorescence for the associated well normalized to the fluorescence of the passive reference dye (ROX). Values are means ± SD (two-sided *t* test, n = 3,) ns, no significant. Source data are provided as a Source Data file.

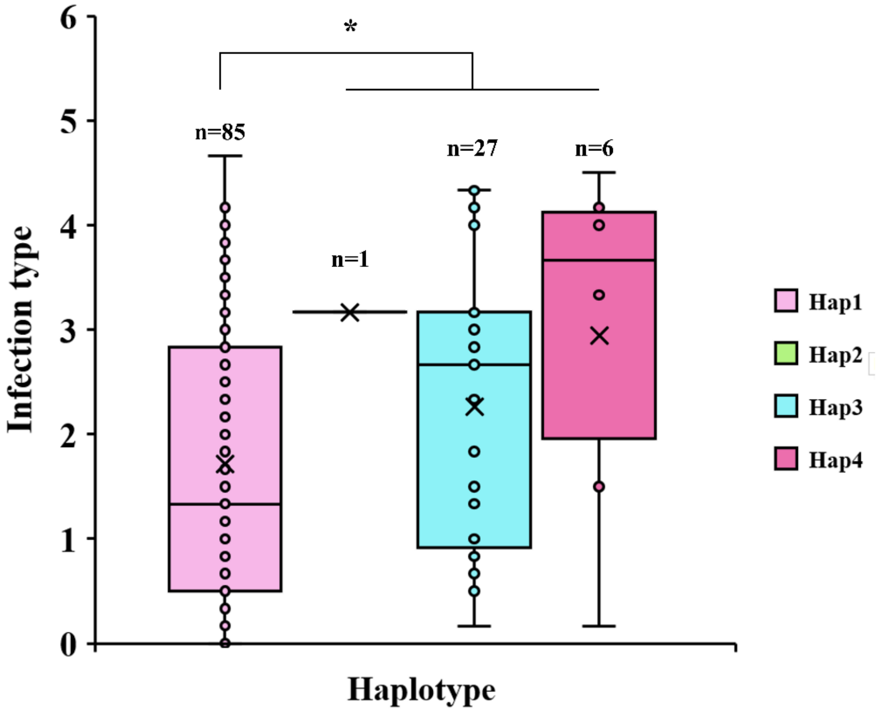

Supplementary Figure 10. Comparison of WYMV resistance between an independent set of varieties carrying Hap 1-4 genotypes. The infection type is derived from the mean value of all replicates. n represent the number of wheat accessions with the corresponding haplotype. Statistics: for both datasets, two-sided *t* test was performed, $P = 0.0159$, * $P < 0.05$. Source data are provided as a Source Data file.

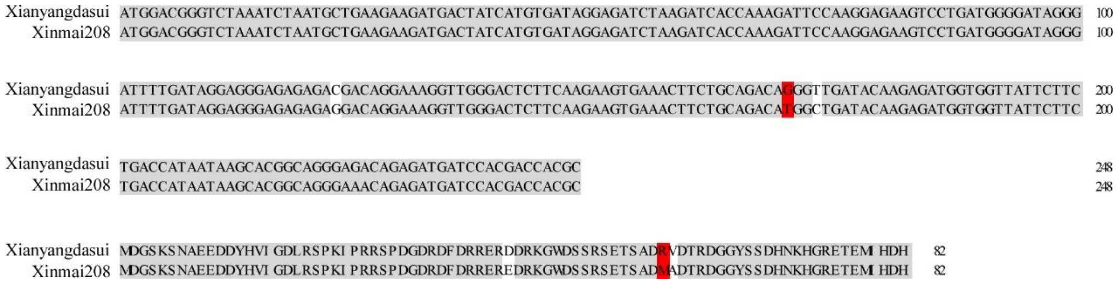

Supplementary Figure 11. sequence alignment analysis of *TaMTB*$^{1-288}$ and TaMTB$^{1\text{-}82aa}$ in cv. Xianyangdasui and cv. Xinmai208. SNP176A/C (reverse complementary) and corresponding amino acid variation was marked in red.

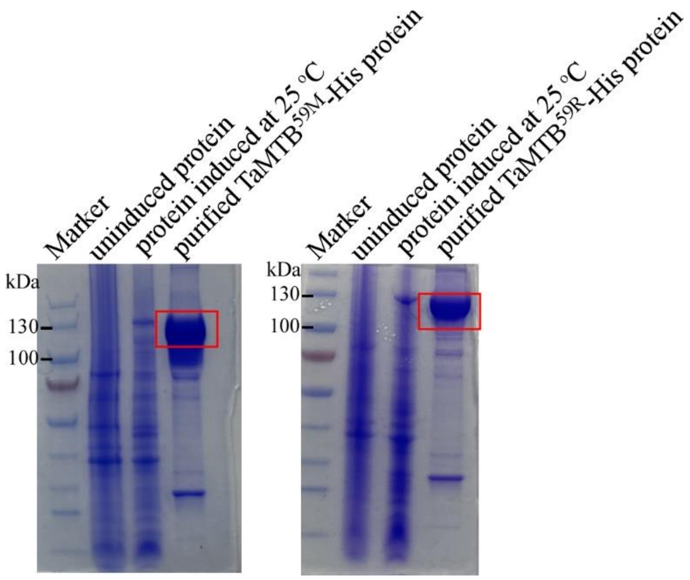

Supplementary Figure 12. Coomassie blue staining of purified proteins. TaMTB^{59M} represents TaMTB(SNP176A) and TaMTB^{59R} represents TaMTB(SNP176C). The red box shows the target protein. Three times each experiment was repeated independently with similar results. Source data are provided as a Source Data file.

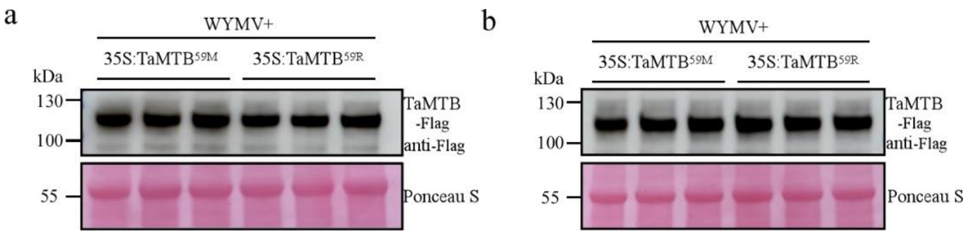

Supplementary Figure 13. Detection of TaMTB protein in assayed plants. a TaMTB expression in 35S:TaMTB(SNP176A) + WYMV and 35S:TaMTB(SNP176C) +WYMV inoculated wheat plants were detected by western blot using a Flag-specific antibody. **b** TaMTB expression in 35S:TaMTB(SNP176A) + 35S:CP and 35S:TaMTB(SNP176C) + 35S:CP inoculated wheat plants were detected by western blot using a Flag-specific antibody. TaMTB^{59M} represents TaMTB(SNP176A) and TaMTB^{59R} represents TaMTB(SNP176C). Three times each experiment was repeated independently with similar results. Source data are provided as a Source Data file.

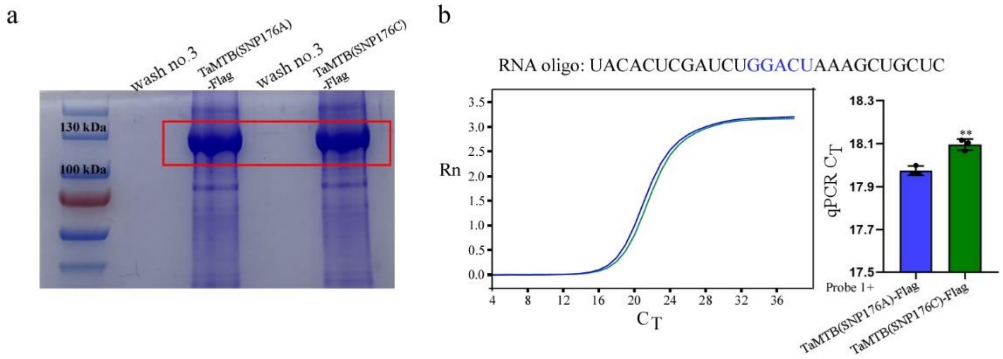

Supplementary Figure 14. Methyltransferase activity detection of TaMTB(SNP176A/C). a Coomassie blue staining of purified IP-TaMTB(SNP176A) and IP-TaMTB(SNP176C). The red box shows the target protein. **b** Real-time fluorescence amplification curves and bar plot of the threshold cycle (CT) of qPCR showing SELECT results for detecting m^{6}A level of RNA oligo with TaMTB(SNP176A) and TaMTB(SNP176C) treatment, respectively. Values are means ± SD (two-sided t test, n = 3, $P = 0.0076$) **$P < 0.01$. The blue sequence represents the m^{6}A motif. Source data are provided as a Source Data file.

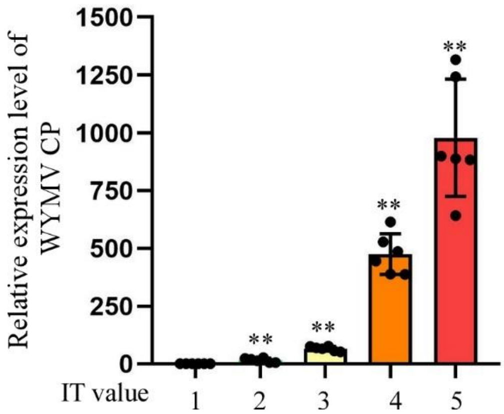

Supplementary Figure 15. Detection for WYMV resistance of these plants which belongs to different infection types (ITs). Relative expression levels of WYMV CP in plants that belongs to different ITs by qRT-PCR. Values are means ± SD (two-sided t test, n = 6, $P = 0.0039$, < 0.0001, < 0.0001, 0.003) **$P < 0.01$. Source data are provided as a Source Data file.

Supplementary Table 1. Detail information of the significant SNPs that detected on 4B.

**SNP**	**Chromosome**	**Position**	***P* value**
AX-110644364	4B	146486803	7.37E-04
AX-110965957	4B	288176992	2.46E-04
AX-108846438	4B	534335201	9.95E-04
AX-95628955	4B	552031373	9.10E-04
AX-94770974	4B	581078205	5.09E-04
AX-94684920	4B	581078279	5.09E-04
AX-108992830	4B	582225014	3.38E-04
TraesCS4B02G314000_602961772	4B	602961772	8.90E-04
TraesCS4B02G314000_602961851	4B	602961851	7.42E-04
TraesCS4B02G314000_602962010	4B	602962010	1.57E-04
TraesCS4B02G314000_602964440	4B	602964440	8.47E-05
TraesCS4B02G314000_602966704	4B	602966704	1.71E-04
TraesCS4B02G314000_SNP208	4B	602966833	5.80E-04
TraesCS4B02G314000_SNP1012	4B	602967623	5.80E-04
TraesCS4B02G314000_SNP1479	4B	602968104	5.80E-04
TraesCS4B02G314000_SNP2481	4B	602968112	5.80E-04
TraesCS4B02G314000_SNP2537	4B	602968136	7.86E-04
TraesCS4B02G314000_SNP3928	4B	602968207	3.53E-05
TraesCS4B02G314000_602969295	4B	602969295	5.80E-04
TraesCS4B03G0816600_602969863	4B	602969863	5.80E-04
TraesCS4B03G0816600_602970075	4B	602970075	5.80E-04
AX-94802309	4B	603038015	5.95E-05
AX-111829807	4B	603046808	2.43E-04
AX-109455736	4B	603981906	4.40E-04
AX-109058948	4B	609108950	5.39E-04
AX-108729855	4B	609163952	6.60E-04
AX-95628759	4B	609513136	7.96E-04
AX-111114716	4B	610226630	9.42E-04

Supplementary Table 2. Nucleotide localization and enrichment of the m^{6}A peaks identified in WYMV genomics by m^{6}A-seq.

m6A peaks number	Gene region	Start site	End site	Fold enrichment
1	RNA1 CDS	2854	3401	1.54888
2	RNA1 CDS	3681	4104	8.09
3	RNA1 CDS	6600	6990	5.20
4	RNA1 CDS	7273	7620	5.75
5	RNA2 3'UTR	3335	3628	4.03219

Supplementary Table 3. Information of predicted m^{6}A modification sites.

Strand	Position	Sequence context	Annotation	Score	Decision
RNA2	3538	TCGAGCCAAGGGACACCTGCATAGA	3'UTR	0.602	Moderate confidenc
RNA1	3956	AUCCCAACAAGGACAUGCACUCAGU	CDS	0.617	Moderate confidenc
RNA1	4091	CCCACAGAAUGGACAUAUCAACCCA	CDS	0.648	High confidenc
RNA1	6777	CGUCAACGCAGGACUAAAGCUUCGA	CDS	0.659	High confidenc
gRNA1	6800	GAAUCUCAAUGGACAAAUUAAAGAG	CDS	0.621	High confidenc
RNA1	7368	UGGACACCCAGAACUAACCUAAACC	CDS	0.595	Moderate confidenc

Note: the predicted m^{6}A modification site was marked with red.
