# Supplementary material for: N6-methyladenosine RNA modification promotes viral genomic RNA stability and infection
Source: Nat Commun. 2022 Nov 2;13:6576. doi: 10.1038/s41467-022-34362-x (PMC9629889; doi:10.1038/s41467-022-34362-x)
Supplement: Supplementary file 2 — Description of Additional Supplementary Files [file 41467_2022_34362_MOESM2_ESM.pdf]

Description of Additional Supplementary Files

File Name: Supplementary Data 1
Description: 31 candidate genes identified in bulked transcriptome sequencing of resistant and susceptible lines.

File Name: Supplementary Data 2
Description: Non-synonymous mutation information of nine candidate genes in the associated population.

File Name: Supplementary Data 3
Description: The primers used in this study.
