# Supplementary material for: N6-methyladenosine RNA modification promotes viral genomic RNA stability and infection
Source: Nat Commun. 2022 Nov 2;13:6576. doi: 10.1038/s41467-022-34362-x (PMC9629889; doi:10.1038/s41467-022-34362-x)
Supplement: Supplementary file 6 — Reporting Summary [file 41467_2022_34362_MOESM6_ESM.pdf]

nature portfolio

Corresponding author(s): Jian Yang

Last updated by author(s): 2022.10.17

Reporting Summary

Nature Portfolio wishes to improve the reproducibility of the work that we publish. This form provides structure for consistency and transparency in reporting. For further information on Nature Portfolio policies, see our Editorial Policies and the Editorial Policy Checklist.

Statistics

For all statistical analyses, confirm that the following items are present in the figure legend, table legend, main text, or Methods section.

n/a	Confirmed	
☐	☒	The exact sample size (*n*) for each experimental group/condition, given as a discrete number and unit of measurement
☐	☒	A statement on whether measurements were taken from distinct samples or whether the same sample was measured repeatedly
☐	☒	The statistical test(s) used AND whether they are one- or two-sided<br>*Only common tests should be described solely by name; describe more complex techniques in the Methods section.*
☒	☐	A description of all covariates tested
☒	☐	A description of any assumptions or corrections, such as tests of normality and adjustment for multiple comparisons
☒	☐	A full description of the statistical parameters including central tendency (e.g. means) or other basic estimates (e.g. regression coefficient) AND variation (e.g. standard deviation) or associated estimates of uncertainty (e.g. confidence intervals)
☒	☐	For null hypothesis testing, the test statistic (e.g. *F*, *t*, *r*) with confidence intervals, effect sizes, degrees of freedom and *P* value noted<br>*Give P values as exact values whenever suitable.*
☒	☐	For Bayesian analysis, information on the choice of priors and Markov chain Monte Carlo settings
☒	☐	For hierarchical and complex designs, identification of the appropriate level for tests and full reporting of outcomes
☒	☐	Estimates of effect sizes (e.g. Cohen's *d*, Pearson's *r*), indicating how they were calculated

Our web collection on statistics for biologists contains articles on many of the points above.

Software and code

Policy information about availability of computer code

Data collection	Nanotemper NT.115<br>Confocal microscopic images were aquired with a TCS SP8 X confocal microscopy.<br>ThermoFisher QuantStudio 5 for qPCR data collection and analysis.
Data analysis	1. GWAS analysis was implemented using GAPIT Version 3 packages in R software based on the mixed linear model (PCA+K)<br>2. QTL IciMapping V4.1 (https://isbreeding.caas.cn/rj/qtllcmapping/294444.htm).<br>3.Mo.Affinity Analysis v2.3<br>4.MEGA 7.0 software<br>5.Microsoft excel.<br>6.GraphPad Prism 8.<br>7.MO.Affinity Analysis v2.3

For manuscripts utilizing custom algorithms or software that are central to the research but not yet described in published literature, software must be made available to editors and reviewers. We strongly encourage code deposition in a community repository (e.g. GitHub). See the Nature Portfolio guidelines for submitting code & software for further information.

Data

Policy information about availability of data

All manuscripts must include a data availability statement. This statement should provide the following information, where applicable:

- Accession codes, unique identifiers, or web links for publicly available datasets
- A description of any restrictions on data availability
- For clinical datasets or third party data, please ensure that the statement adheres to our policy

Data supporting the findings of this work are available within the paper and its Supplementary Information files, or from the corresponding author upon request. The raw m6A-seq plus RNA-seq data are available in the NCBI database under accession code PRJNA694346. Source data are provided with this paper.

Human research participants

Policy information about studies involving human research participants and Sex and Gender in Research.

Reporting on sex and gender	n/a
Population characteristics	n/a
Recruitment	n/a
Ethics oversight	n/a

Note that full information on the approval of the study protocol must also be provided in the manuscript.

Field-specific reporting

Please select the one below that is the best fit for your research. If you are not sure, read the appropriate sections before making your selection.

☒ Life sciences ☐ Behavioural & social sciences ☐ Ecological, evolutionary & environmental sciences

For a reference copy of the document with all sections, see nature.com/documents/nr-reporting-summary-flat.pdf

Life sciences study design

All studies must disclose on these points even when the disclosure is negative.

Sample size	Sample sizes were not predetermined, but chosen to be similar to common sample sizes in previous studies in the field.
Data exclusions	no data were excluded from the analyses
Replication	All attempts at replication were successful. The number of replicates is indicated in the corresponding figure legend and/or in the corresponding material and method section.
Randomization	Randomization was not appropriate for this study.
Blinding	Blinding was not appropriate for this study.

Reporting for specific materials, systems and methods

We require information from authors about some types of materials, experimental systems and methods used in many studies. Here, indicate whether each material, system or method listed is relevant to your study. If you are not sure if a list item applies to your research, read the appropriate section before selecting a response.

Materials & experimental systems

n/a	Involved in the study
☐	☒ Antibodies
☐	☒ Eukaryotic cell lines
☒	☐ Palaeontology and archaeology
☒	☐ Animals and other organisms
☒	☐ Clinical data
☒	☐ Dual use research of concern

Methods

n/a	Involved in the study
☒	☐ ChIP-seq
☒	☐ Flow cytometry
☒	☐ MRI-based neuroimaging

Antibodies

Antibodies used

1.WYMV CP-specific antibody (prepared by Huaan Biotechnology Co., Ltd, Hangzhou, Zhejiang, China, and stored in our lab)2. m6A antibody (Cat. No. 202 003, Synaptic Systems, Goettingen, Germany) 3. anti-Flag and GFP monoclonal antibody (TransGen Biotech, Beijing, China, HT201-01 and HT801-01) 4.Goat Anti-Mouse IgG (Abbkine Scientific Co., Ltd., California, USA, A21010)

Validation

1.WYMV CP-specific antibody (prepared by Huaan Biotechnology Co., Ltd, Hangzhou, Zhejiang, China, and stored in our lab)2. m6A antibody (Cat. No. 202 003, Synaptic Systems, Goettingen, Germany) 3. anti-Flag and GFP monoclonal antibody (TransGen Biotech, Beijing, China, HT201-01 and HT801-01) 4.Goat Anti-Mouse IgG (Abbkine Scientific Co., Ltd., California, USA, A21010)

Eukaryotic cell lines

Policy information about cell lines and Sex and Gender in Research

Cell line source(s)

Primary cultures of Sf9 cells were purchased from Invitrogen and maintained in SFII-900 medium. The Sf9 insect cell line is a clonal isolate derived from the parental Spodoptera frugiperda cell line IPLB-Sf-21-AE.

Authentication

The SF9 cell lines expressed recombinant protein was identified by western blot with specific anti-Flag.

Mycoplasma contamination

all cell lines tested negative for mycoplasma contamination.

Commonly misidentified lines
(See ICLAC register)

No any commonly misidentified lines was used in this study.
